# XPO1 coordinates human cytomegalovirus maturation by modulating genome encapsidation and secondary envelopment

**DOI:** 10.1016/j.cellin.2026.100364

**Published:** 2026-09-09

**Authors:** Jin-Peng Wu, Yue-Peng Zhou, Meng-Jie Mei, Yu-Ting Pan, Jin-Yan Sun, Wen-Bo Zeng, Han Cheng, Min-Hua Luo, Bo Yang

**Affiliations:** a State Key Laboratory of Virology and Biosafety, Wuhan Institute of Virology, Chinese Academy of Sciences, Wuhan, Hubei, 430071, China; b University of Chinese Academy of Sciences, Beijing, 101408, China; c State Key Laboratory of Magnetic Resonance Spectroscopy and Imaging, Innovation Academy for Precision Measurement Science and Technology, Chinese Academy of Sciences, Wuhan, Hubei, 430071, China; d Shanghai Public Health Clinical Center, Fudan University, Shanghai, 2000083, China

**Keywords:** Human cytomegalovirus, Exportin 1, pUL77, Nuclear capsid assembly, Autophagy, Host-directed antiviral therapy

## Abstract

Human cytomegalovirus (HCMV) replication depends on tightly coordinated nuclear capsid assembly and cytoplasmic nucleocapsid maturation, yet host factors controlling these processes remain poorly defined. Here, we identify exportin 1 (XPO1) as a dual-phase regulator that coordinates HCMV maturation from nucleus to cytoplasm. XPO1 inhibition induces aberrant nuclear electron-dense speckles and accumulation of B-capsids. Mechanistically, nuclear retention of pp65 drives speckle formation but does not account for B-capsid accumulation. XPO1 recognizes a functional nuclear export signal (NES) within pUL77 and regulates its proper intranuclear localization. Inhibiting XPO1 or disrupting the NES induces intranuclear pUL77 aggregation and B-capsid accumulation. XPO1 is also required for viral assembly compartment formation and maintenance of late-stage autophagic flux, both of which are essential for secondary envelopment. Our findings highlight the key role of XPO1 in virion maturation and the therapeutic potential of repurposing FDA-approved XPO1 inhibitors as a host-directed antiviral strategy.

## Highlights

• XPO1 is essential for efficient HCMV replication.
• XPO1 regulates pUL77 nuclear localization to facilitate viral genome encapsidation.
• XPO1 links autophagy to cytoplasmic envelopment of HCMV.
• FDA-approved XPO1 inhibitor selinexor exhibits potent anti-HCMV activity.

## Graphical abstract

**Figure:**
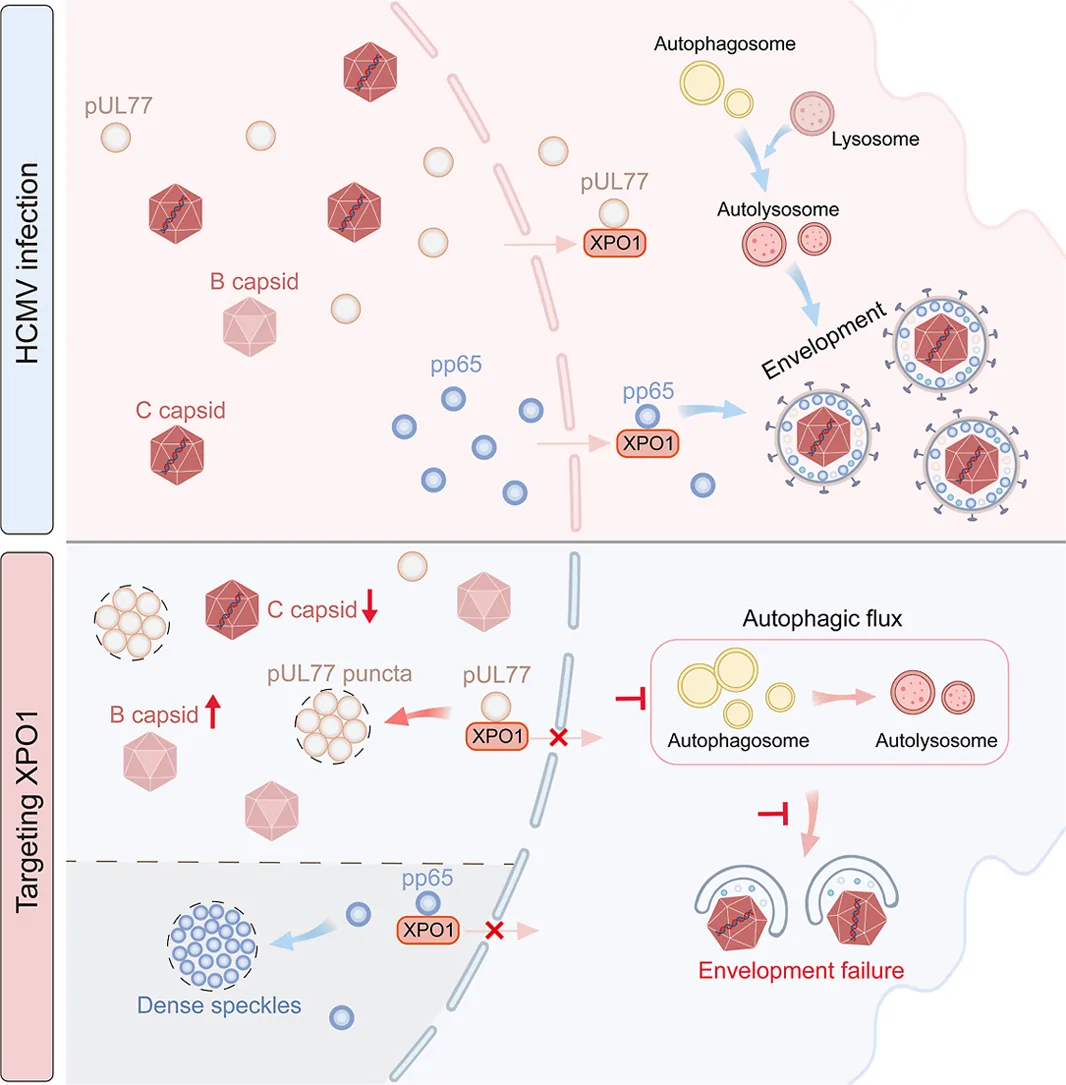

## 1 Introduction

Human cytomegalovirus (HCMV) belongs to the *Beta-herpesvirinae* subfamily and is a highly prevalent human pathogen that infects more than 60% of the global population by adulthood (Zuhair et al., 2019). Primary HCMV infection is typically asymptomatic in healthy individuals, yet the virus establishes lifelong latency and reactivates when immune surveillance is impaired (Wang & Zhao, 2020). In immunocompromised patients, including those with HIV/AIDS and organ transplant recipients, HCMV can cause severe complications such as pneumonitis, retinitis, hepatitis, and graft-versus-host disease (Griffiths & Reeves, 2021). HCMV is also the leading viral cause of congenital birth defects (Kirby, 2016). The developing fetal brain and immature immune system render neonates particularly susceptible to congenital HCMV infection, which causes sensorineural hearing loss, ocular disease, neurodevelopmental impairment, and other congenital abnormalities (Reddy et al., 2026). Despite several decades of research, no effective vaccine for HCMV is currently available. Clinical management relies exclusively on a limited choice of antivirals, primarily ganciclovir and valganciclovir, targeting the viral DNA polymerase (Modrzejewska et al., 2024; Singh et al., 2020). However, these frontline therapies are frequently undermined by hematological toxicity, renal impairment, and the rapid emergence of drug-resistant variants (Lurain & Chou, 2010; Raglow & Kaul, 2023). These clinical limitations underscore an urgent need to identify novel host- and virus-specific targets for innovative antiviral strategies.

HCMV virion assembly and maturation have recently emerged as promising antiviral targets, as demonstrated by the clinical success of letermovir (terminase complex inhibitor, approved in 2017 for prophylaxis) and maribavir (pUL97 kinase inhibitor, approved in 2021 for refractory disease) (Khawaja et al., 2025). However, letermovir- and maribavir-resistant HCMV variants have been detected within weeks to months of therapy (Razonable, 2024), highlighting a fundamental limitation of therapies that rely solely on viral protein targets. Thus, targeting host factors essential for virion assembly has emerged as a compelling alternative strategy that could circumvent existing resistance mechanisms.

HCMV virion maturation is a tightly coordinated, multistep process orchestrated by numerous viral and host proteins (Tyl et al., 2022). In the nucleus, linear viral genomes are circularized and amplified into concatemeric replication intermediates, which are processed by the terminase complex (pUL51/pUL56/pUL89) to generate unit-length genomes (Muller et al., 2021). These genomes are packaged into procapsids via a portal-mediated mechanism, with internal scaffolding proteins removed and packaging completion signaled by the capsid vertex-specific components (CVSC: pUL93/pUL77/pUL48) (Borst et al., 2016; Li et al., 2021, 2023). Accordingly, three types of capsids are generated, reflecting the success or failure of viral genome packaging: A-capsids are empty, lacking both scaffolding and viral genome; B-capsids contain scaffolding but no genome and represent packaging-arrested intermediates; and C-capsids contain viral genome without scaffolding and correspond to mature nucleocapsids (Tandon & Mocarski, 2012). Capsids exit the nucleus via the nuclear egress complex, formed by pUL53/pUL50 and several host factors such as WDR5, p32, lamin-binding proteins, and protein kinase C, which mediate primary envelopment and de-envelopment (Marschall et al., 2017; Tillmanns et al., 2024; Yang et al., 2021). In the cytoplasm, nucleocapsids are trafficked to the viral assembly compartment (vAC), where they acquire tegument proteins and envelope membranes through secondary envelopment (Alwine, 2012). Recent studies have shown that HCMV hijacks host Rab GTPases, vacuolar ATPase components, and exosome-related machinery to facilitate late-stage virion assembly and release (Indran & Britt, 2011; Pavelin et al., 2017; Turner et al., 2020). These studies have identified host factors involved in nuclear egress and subsequent cytoplasmic maturation, but nuclear capsid assembly of HCMV has long been viewed as a predominantly virus-driven process, and host factors that regulate early nuclear capsid assembly remain largely undefined.

The nuclear export receptor exportin 1 (XPO1) has recently emerged as a key regulator of viral assembly for several viruses (Mghezzi-Habellah et al., 2023). XPO1 mediates the Ran-GTP-dependent export of leucine-rich nuclear export signal (NES)-containing proteins and certain RNAs from the nucleus to the cytoplasm (Azmi et al., 2021). This essential host pathway is frequently hijacked by viruses to translocate critical viral proteins and thereby facilitate virion assembly (Mathew et al., 2021). HIV Rev and influenza NS2 exploit XPO1 to shuttle viral ribonucleoprotein complexes to cytoplasmic sites of assembly, and the respiratory syncytial virus matrix protein undergoes an XPO1-dependent nuclear-to-cytoplasmic switch to enable virion formation (Mathew et al., 2021; Neumann et al., 2000; Smith et al., 2025). In HCMV, the tegument proteins pp65 and pUL94 contain functional nuclear localization signals (NLS) and NES motifs and are shuttled from the nucleus to the cytoplasm in an XPO1-dependent manner (Liu et al., 2012; Sanchez et al., 2007). These findings highlight XPO1 as a central node that links the nuclear- and cytoplasmic-phases of virion assembly.

In this study, we demonstrate that XPO1 is indispensable for efficient HCMV replication and functions at multiple stages of the viral life cycle. XPO1 inhibition blocks HCMV replication, triggering the aberrant formation of nuclear electron-dense speckles and accumulation of B-capsids, marking the failure of viral genome encapsidation. Mechanistic studies demonstrate that pp65 is responsible for the formation of nuclear electron-dense speckles, but not for B-capsid accumulation. Further screening revealed that pUL77 is a specific cargo of XPO1, and that its proper intranuclear localization is essential for nuclear capsid maturation. In addition, XPO1 inhibition impairs HCMV secondary envelopment in the cytoplasm, as XPO1 is required to maintain late-stage autophagic flux that supports this process. Together, these findings identify XPO1 as a crucial host regulator of virion assembly across multiple steps and cellular compartments, and highlight it as a promising target for host-directed antiviral strategies against HCMV.

## 2 Results

### 2.1 XPO1 is required for productive HCMV infection

To determine whether XPO1 is required for HCMV replication, we silenced XPO1 expression in human foreskin fibroblasts (HFFs) using RNA interference. Two short hairpin RNAs (shRNAs) targeting XPO1 were generated, resulting in 28% (shXPO1-1) and 92% (shXPO1-2) reductions in XPO1 protein levels, respectively (Fig. 1(A)). Given its higher knockdown efficiency, shXPO1-2 was used for subsequent experiments. HFFs transduced with shXPO1-2 or a scrambled control were infected with HCMV, and viral titers in the supernatants were quantified at 96 h post-infection (hpi) by plaque assay. XPO1 knockdown reduced viral titers by approximately 77% compared with controls (Fig. 1(B)), indicating that XPO1 contributes to efficient HCMV replication. To further validate the role of XPO1 in HCMV infection, we treated infected HFFs with two selective XPO1 inhibitors KPT330 and KPT335. Both compounds exhibited potent antiviral activity, with half-maximal inhibitory concentration (IC_50_) values of 10 nM and 20 nM and selectivity indices (SI) of 31.5 and 18.7, respectively (Fig. 1(C)). These data collectively indicate that XPO1 is required for efficient HCMV replication.

**Fig. 1:**
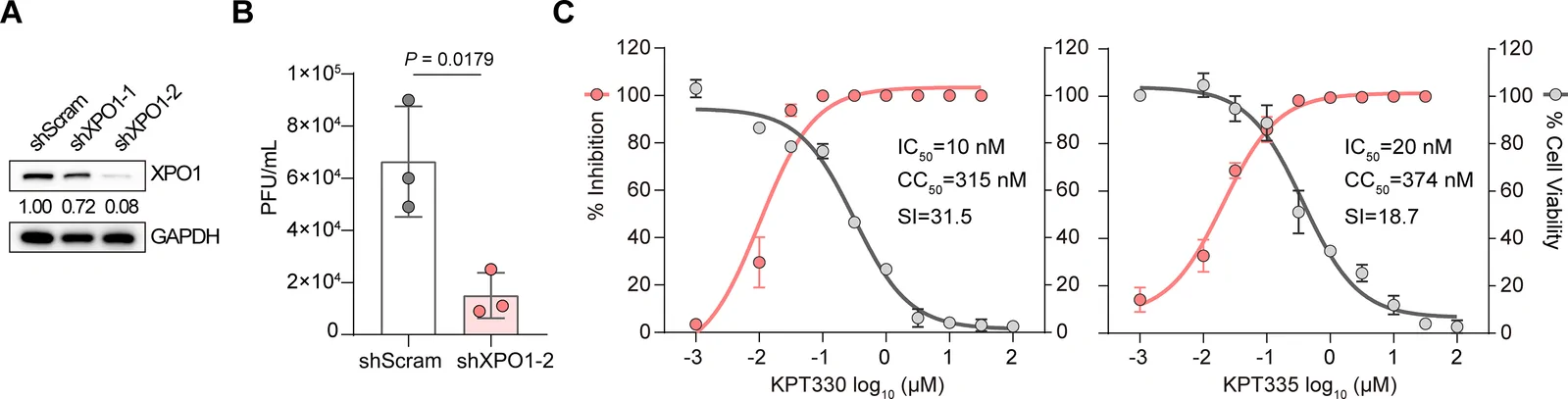
**XPO1 depletion or pharmacological inhibition attenuates HCMV replication.** **(A)** Efficiency of XPO1 knockdown. Human foreskin fibroblasts (HFFs) were transduced with lentiviruses expressing scrambled shRNA (shScram) or two XPO1-targeting shRNAs (shXPO1-1, shXPO1-2) and selected with puromycin (1 μg/mL) for 5 days. XPO1 protein levels were assessed by immunoblotting. Relative XPO1 expression, normalized to GAPDH, is indicated below the respective bands. **(B)** XPO1 knockdown attenuates HCMV replication. shRNA-transduced HFFs were infected with HCMV at a multiplicity of infection (MOI) of 1. Supernatants were collected at 96 hpi, and viral titers were determined by plaque assay. **(C)** Antiviral potency and cytotoxicity of XPO1 inhibitors. To determine the half-maximal inhibitory concentration (IC_50_), HFFs were infected with HCMV (MOI = 1) and treated with serial dilutions of KPT330 or KPT335. Supernatants were collected at 96 hpi for viral titration. The 50% cytotoxic concentration (CC_50_) was determined in HFFs at 96 h post-treatment using a 3-(4,5-dimethylthiazol-2-yl)-2,5-diphenyltetrazolium bromide (MTT) assay. Data were collected from three independent experiments, and results are presented as mean ± SD. Statistical significance was determined by a two-tailed unpaired *t*-test.

### 2.2 XPO1 inhibition induces nuclear dense speckle formation and B-capsid accumulation

To identify the stage of the viral life cycle affected by XPO1 inhibition, we first examined the expression of representative immediate-early (IE1/2), early (pUL44), and late (pp28) viral proteins. XPO1 inhibition did not noticeably alter the levels of these proteins (Fig. 2(A)), suggesting that the impact of inhibition of XPO1 expression on virus replication could not be explained by a decrease in viral protein synthesis.

**Fig. 2:**
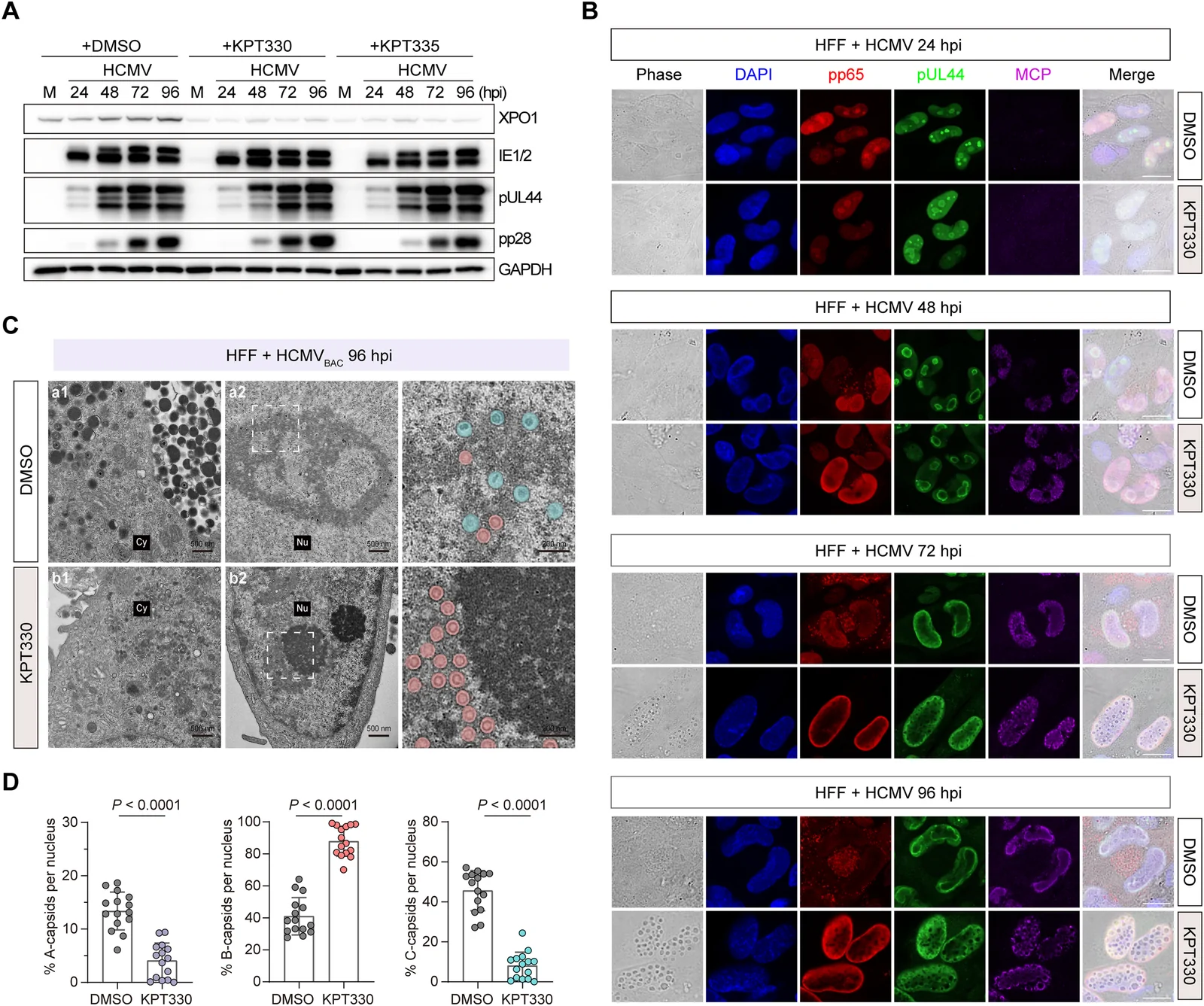
**XPO1 inhibition induces nuclear dense speckle formation and impairs nuclear capsid maturation.** **(A)** Kinetics of viral protein expression. Infected HFFs (MOI = 1) were treated with DMSO, KPT330 (0.02 μM), or KPT335 (0.02 μM), and harvested at the indicated times for immunoblot analysis of XPO1, IE1/2, pUL44, and pp28. **(B)** Spatiotemporal organization of nuclear viral replication compartments (vRCs). HFFs were infected (MOI = 1) and treated with KPT330 (0.02 μM) or DMSO vehicle. Cells were fixed at the indicated times, and stained with antibodies against MCP (purple), pp65 (red), and the vRC marker pUL44 (green). Nuclei were counterstained with DAPI (blue). Images are representative of three independent experiments. Scale bars, 20 μm. **(C)** Ultrastructural analysis of nuclear dense speckles. HFFs were infected with HCMV_BAC_ (MOI = 1) and fixed at 96 hpi for transmission electron microscopy (TEM). Electron micrographs of a1 and a2 show the cytoplasmic and nuclear regions, respectively, from the same cell. Panels b1 and b2 are likewise derived from the same cell. Regions within the white dashed boxes are shown enlarged in the right panel. C-capsids are highlighted in cyan and B-capsids in pink. Scale bars, 500 nm (main) and 200 nm (enlarged images). Nu, nucleus; Cy, cytoplasm. **(D)** Quantitative analysis of nuclear capsid populations. The relative proportions of A-, B-, and C-capsids were determined from fifteen nuclei per condition. Data are represented as mean ± SD. *P* values were calculated using the Mann–Whitney test.

We next assessed the formation of viral replication compartments (vRCs) by staining the DNA polymerase subunit pUL44, a marker of vRC (Strang et al., 2012). In both control and KPT330-treated cells, pUL44 progressed from multiple small foci (24 hpi), bipolar foci (48 hpi), to single large nuclear vRCs by 72 hpi and 96 hpi (Fig. 2(B)), indicating that XPO1 is not required for vRC formation. Although the subnuclear localization of pUL44 appeared unaffected, XPO1 inhibition caused a striking redistribution of major capsid protein (MCP) within the nucleus at 72 and 96 hpi, coincident with the emergence of numerous phase-dense spherical bodies in infected nuclei (Fig. 2(B)). Ultrastructural analysis via transmission electron microscopy (TEM) revealed that these phase-dense spherical bodies consisted of large electron-dense granular aggregates, and that these nuclear dense speckles were often surrounded by B-capsids (Fig. 2(C)).

We further evaluated capsid formation in control HFFs and cells with XPO1 inhibition. Quantitative analysis revealed that KPT330 treatment increased the proportion of B-capsids (from 41% to 88%) while reducing the proportions of A-capsids (from 13% to 4%) and C-capsids (from 46% to 8%) in HCMV_BAC_-infected cells (Fig. 2(D)). Similar defects were observed with KPT335 treatment and XPO1 depletion (Fig. S1). These results indicate that XPO1 inhibition induces the formation of aberrant nuclear aggregates and disrupts nuclear capsid maturation.

### 2.3 HCMV pp65 nuclear retention drives dense speckle formation independently of the capsid maturation defect

Because XPO1 mediates nuclear export, we hypothesized that its inhibition would cause nuclear retention of viral proteins and thereby promote the formation of the nuclear dense speckles, which may disrupt capsid maturation. We first examined the localization of the well-characterized tegument protein pp65, which functions in the nucleus early during infection and later shuttles to the cytoplasm for virion assembly in an XPO1-dependent manner (Sanchez et al., 2007). In control cells, pp65 redistributed from a predominantly nuclear localization at early time to a cytoplasmic localization, accumulating in the vAC at later stage of infection, whereas in XPO1-inhibited cells, pp65 remained largely confined to the nucleus (Fig. S2A). Intriguingly, pp65 did not appear to localize within the dense spherical bodies (Fig. S2A). We reasoned that the densely packed proteins within these aggregates might cause epitope masking, thereby preventing antibody access. To address this, we expressed pp65-mScarlet in HCMV_BAC_-infected HFFs followed by treatment with KPT330 or DMSO, and found that pp65-mScarlet localized exclusively to nuclear phase-dense bodies in KPT330-treated cells (Fig. S2B). These data suggest that XPO1 inhibition promotes pp65 nuclear aggregation, which colocalizes with the nuclear phase-dense inclusions.

To test whether pp65 nuclear retention drives nuclear dense speckle formation, we generated a pp65-deficient Towne BAC variant (HCMV_dpp65_) (Fig. S3A). As expected, no pp65 expression was detected in HCMV_dpp65_-infected HFFs (Fig. S3B). Upon KPT330 treatment, the characteristic nuclear phase-dense spherical bodies were entirely absent in HCMV_dpp65_-infected cells (Fig. S3C). Ultrastructural analysis confirmed the disappearance of nuclear dense speckles in HCMV_dpp65_-infected cells (Fig. S3D). These findings demonstrate that pp65 nuclear accumulation mediates the formation of the nuclear dense speckles.

However, KPT330 still caused nuclear accumulation of B-capsids and reduced the proportions of A- and C-capsids in HCMV_dpp65_-infected nuclei, similar to the effects observed in parental HCMV_BAC_-infected cells (Fig. S3E). Consistent with this observation, KPT330 inhibited replication of HCMV_dpp65_ and HCMV_BAC_ to a similar extent in HFFs (Fig. S3F). Together, these results demonstrate that while XPO1 inhibition causes pp65 nuclear retention and consequent formation of nuclear dense speckles, neither of these events contributes to the observed capsid maturation defect.

### 2.4 HCMV pUL77 nuclear distribution is responsive to XPO1 inhibition

B-capsids are the scaffold-containing, DNA-devoid intermediate capsids that assemble in the nucleus prior to viral genome packaging. Therefore, B-capsid accumulation upon XPO1 inhibition suggested a deficit in genome encapsidation. Because this B-capsid buildup is unrelated to pp65-mediated nuclear aggregate formation, we next investigated whether XPO1 inhibition affects components of the essential viral genome packaging machinery.

We first tested whether XPO1 interacts with key viral proteins (pUL51, pUL56, pUL77, pUL89, and pUL93) involved in genome packaging. Myc-tagged XPO1 was co-expressed with FLAG-tagged viral proteins in HEK293T cells. Co-immunoprecipitation analysis revealed that pUL77 and pUL89 robustly co-precipitated with XPO1 (Fig. 3(A)), whereas pUL56 and pUL93 showed only modest enrichment in the immunoprecipitates. We then examined whether XPO1 inhibition alters the subnuclear distribution of these viral proteins. mScarlet-tagged pUL77, pUL89, and pUL93 were expressed in HFFs, followed by HCMV infection and KPT330 treatment. For pUL56, a recombinant HCMV expressing Myc-tagged pUL56 was created and used (Fig. S4A). No significant changes in the distribution of pUL56, pUL89, or pUL93 were observed upon XPO1 inhibition. However, pUL77 was markedly redistributed from its typical diffuse nuclear pattern into discrete puncta (Fig. S4B–E). These data suggest that pUL77 localization was dependent on XPO1-mediated transport or spatial regulation.

**Fig. 3:**
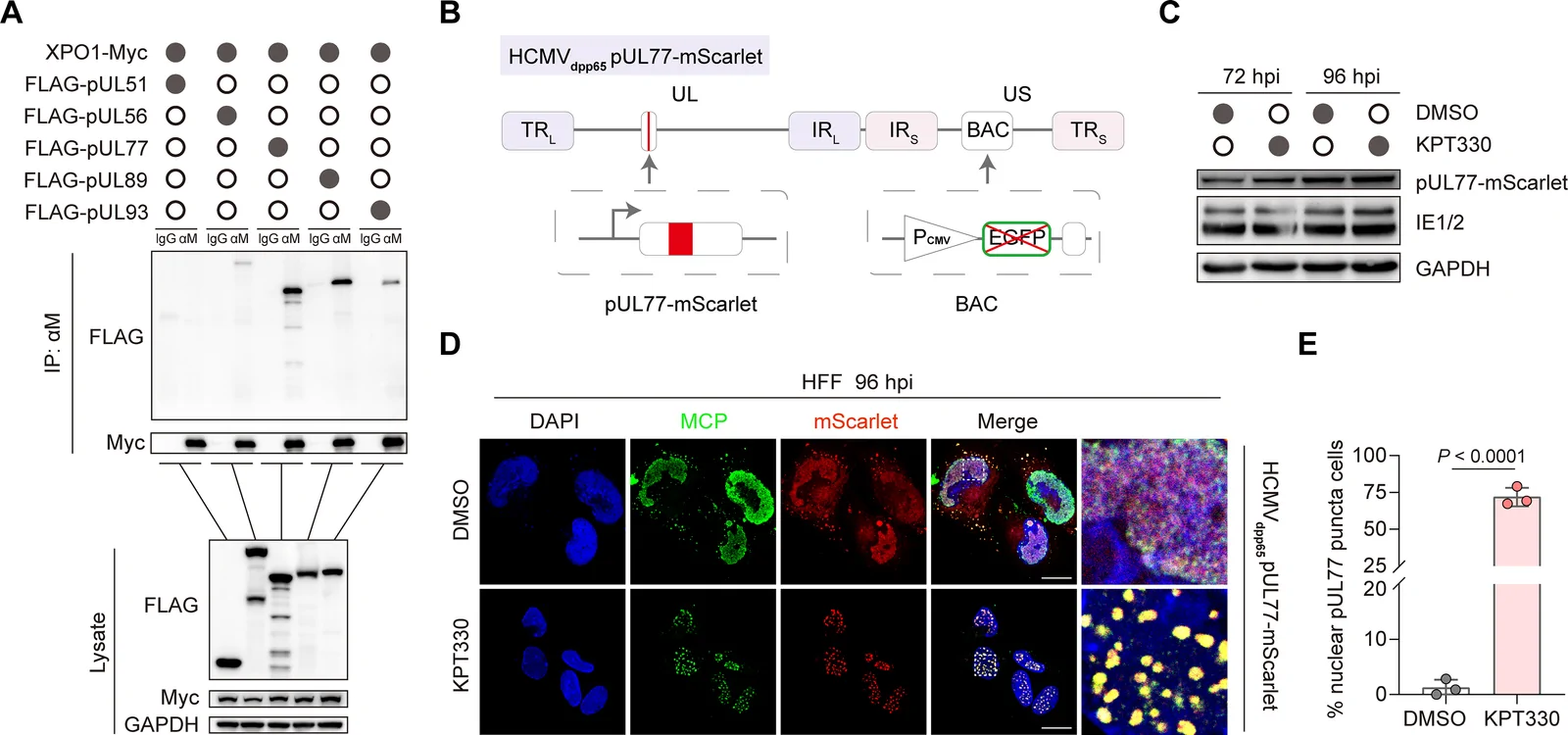
**XPO1 interacts with pUL77 and regulates its intranuclear distribution.** **(A)** Screening of viral proteins involved in genome encapsidation for XPO1 interactions. 293T cells were co-transfected with constructs expressing Myc-XPO1 and FLAG-tagged viral protein candidates (pUL51, pUL56, pUL77, pUL89, or pUL93). At 48 h post-transfection, cells were harvested and cell lysates were subjected to immunoprecipitation with an anti-Myc monoclonal antibody (αM) or control IgG, followed by immunoblotting with the indicated antibodies. **(B)** Schematic genome diagram of HCMV_dpp65_ pUL77-mScarlet. The EGFP cassette was removed from the HCMV_dpp65_, and the mScarlet cassette was inserted at amino acid 154 of pUL77 via homologous recombination to generate the recombinant virus HCMV_dpp65_ pUL77-mScarlet. **(C)** Effect of KPT330 on pUL77-mScarlet expression. HFFs were infected with HCMV_dpp65_ pUL77-mScarlet (MOI = 1), and treated with KPT330 or DMSO. At 72 hpi and 96 hpi, cells were harvested and cell lysates were subjected to immunoblotting for pUL77-mScarlet and IE1/2, with GAPDH as a loading control. **(D)** XPO1 inhibition induces pUL77 nuclear puncta formation. HFFs were infected with the HCMV_dpp65_ pUL77-mScarlet at an MOI of 1, followed by treatment with KPT330 or DMSO. Cells were fixed at 96 hpi, and the localization of MCP (green) and pUL77-mScarlet (red) was assessed by immunofluorescence assay (IFA). Nuclei were counterstained with DAPI (blue). Representative images from three independent experiments are shown. Scale bars, 20 μm. **(E)** Quantitative analysis of punctate pUL77 distribution. The percentage of cells exhibiting nuclear punctate pUL77 (panel D) was further quantified (>200 cells per treatment group). Data were collected from three independent experiments, and results are presented as mean ± SD. *P* value was calculated using two-tailed unpaired *t*-test.

To further confirm this phenotype, we constructed a virus variant expressing mScarlet-tagged pUL77 in the HCMV_dpp65_ BAC background, which also ensured this phenotype was not an artifact of pp65 nuclear retention (Fig. 3(B)). As expected, KPT330 treatment did not affect pUL77 expression (Fig. 3(C)), but triggered the formation of nuclear pUL77-mScarlet puncta (Fig. 3(D)). Notably, these puncta colocalized with the aberrant MCP aggregates described above. Quantitative image analysis showed that this redistribution occurred in approximately 75% of infected cells (Fig. 3(E)). Collectively, these findings suggest that XPO1 is required for the nuclear distribution of pUL77 and for viral genome encapsidation.

### 2.5 Identification of XPO1-binding NES in pUL77

To further characterize the XPO1-pUL77 interaction, we mapped the sequence motifs within pUL77 required for XPO1-mediated nuclear export. First, we predicted candidate motifs using the NES prediction tools LocNES and Wregex (Table S1). To functionally validate these candidate NESs, we performed a nuclear export assay using the Rev dNES-mScarlet reporter, which accumulates in the nucleus unless fused to a functional NES (Yang et al., 2022). Since the predicted NESs spanned nearly the entire pUL77 coding sequence, we generated six fragments (∼70–110 a each) and fused them individually to the reporter (Fig. 4(A)). Among them, only fragment 6 (amino acid residues 566–635) fully restored cytoplasmic localization, comparable to that of the positive control Rev-mScarlet (Fig. 4(B)). This region contains the consensus motif _591_LGRLLSVTL_599_, which scored highly by prediction tools and was 100% conserved across examined experimental and clinical HCMV isolates (Fig. 4(C)). We further utilized AlphaFold3 to model the interaction between this motif and XPO1. Structural modeling predicted that residues _591_LGRLLSVTL_599_ adopt an extended conformation that fits into the hydrophobic NES-binding groove of XPO1, mimicking canonical XPO1 cargo recognition (Fig. 4(D)).

**Fig. 4:**
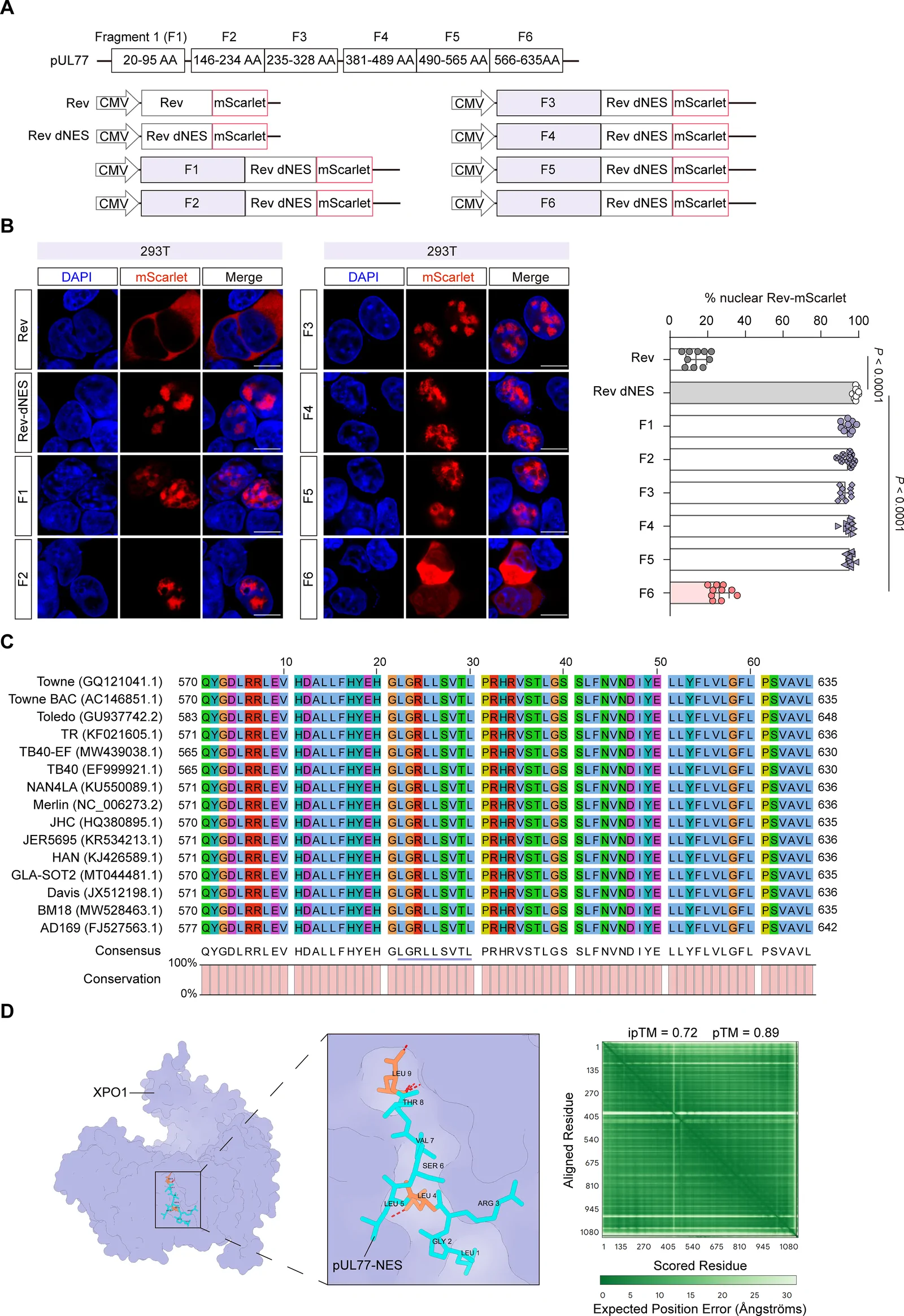
**Mapping of the XPO1-binding NES in pUL77.** **(A)** Schematic diagram of pUL77 fragments fused to the Rev reporter. To map the nuclear export motifs, six fragments of pUL77, each comprising 70–110 amino acids, were fused to a Rev-mScarlet reporter protein lacking its native NES (Rev dNES). **(B)** Subcellular localization of pUL77 fragment-Rev reporters. 293T cells were transfected with plasmids encoding Rev-mScarlet, Rev dNES, or pUL77 fragment-fused reporter proteins. Subcellular localization was analyzed by fluorescence microscopy; nuclei were counterstained with DAPI (blue). Scale bars, 10 μm. Right, quantification of the nucleocytoplasmic distribution of reporter proteins. Data represent the mean ± SD of 10 random fields (>300 cells per group) from three independent experiments. Statistical significance was determined by one-way ANOVA with Dunnett's T3 multiple-comparisons test. **(C)** Sequence alignment of the identified NES region (_591_LGRLLSVTL_599_) across multiple HCMV laboratory and clinical strains. **(D)** Structural modeling of the XPO1–pUL77 NES interaction. Left: Structural prediction of the pUL77 NES peptide (_591_LGRLLSVTL_599_) docked within the hydrophobic groove of XPO1, generated by AlphaFold3. Residues L594 and L599, predicted to make key contacts with XPO1, are highlighted in orange. Right: Predicted aligned error (PAE) per token pair for the predicted model, with a green gradient indicating the PAE value (Å).

### 2.6 HCMV pUL77 NES mutation disrupts viral genome encapsidation

To define how the pUL77 NES controls its nuclear distribution and contributes to the capsid maturation defect caused by XPO1 inhibition, we introduced targeted NES mutations into the HCMV_dpp65_ pUL77 wild-type (WT). Five hydrophobic residues within the NES (_591_LGRLLSVTL_599_) were first substituted with alanine (5A-mut, Fig. 5(A)). This extensive disruption almost completely abolished virus production, as no cytopathic effect was detectable even 14 days after BAC transfection into HFFs (Fig. 5(B)), indicating that severe perturbation of the NES profoundly compromises pUL77 function and HCMV replication.

**Fig. 5:**
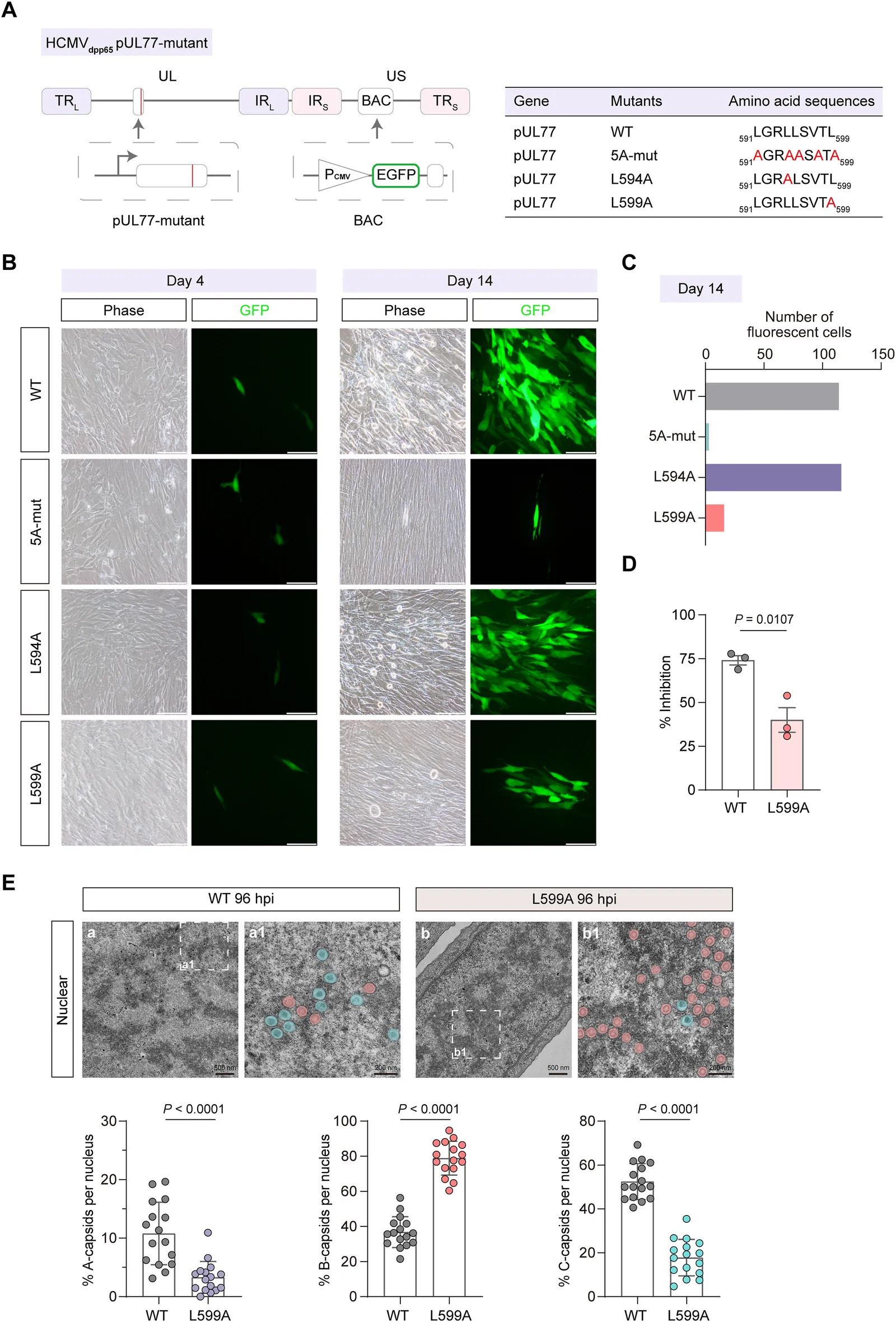
**Mutation of pUL77 NES impairs viral genome encapsidation.** **(A)** Construction of HCMV_dpp65_ pUL77 NES mutants. Schematic genome diagram of pUL77 wild-type (WT) and NES alanine substitution mutants, with the specific residue(s) highlighted in the right table. **(B)** Viral reconstitution and growth. HFFs were transfected with the indicated recombinant BACs and viral reconstitution was monitored on days 4 and 14 post-transfection (dpt) using phase-contrast and fluorescence microscopy. HCMV_dpp65_ with pUL77 WT served as the control. Scale bars, 100 μm. **(C)** Reconstitution efficiency of pUL77 mutants. Quantification of EGFP-positive cells at 14 dpt from the experiments described in (B), reflecting the ability of the mutants to initiate productive infection and spread. **(D)** Antiviral sensitivity of the L599A to XPO1 inhibition. HFFs were infected with HCMV_dpp65_ pUL77 WT or L599A (MOI = 1), and treated with either KPT330 or DMSO. Viral titers were determined at 96 hpi by plaque assay. Results were normalized to the DMSO-treated controls accordingly. Data were collected from three independent experiments, and results are presented as mean ± SEM. *P* value was calculated by two-tailed unpaired *t*-test. **(E)** The pUL77 L599A mutation disrupts viral genome packaging. HFFs were infected with HCMV_dpp65_ pUL77 WT or the mutant L599A (MOI = 1). Cells were fixed at 96 hpi for TEM analysis. C-capsids are highlighted in cyan, and B-capsids in pink. The white dashed box is enlarged in the right panel. Scale bars, 500 nm or 200 nm. Quantification of nuclear capsid populations (*n* = 16 nuclei per condition; error bars represent mean ± SD). *P* values were calculated by the Mann–Whitney test (bottom panel).

To minimize perturbation of viral replication and recover viable mutant viruses for downstream validation, we next focused on individual amino acid residues L594 and L599, which were predicted to contact the hydrophobic groove of XPO1 in our structural modeling (Fig. 4(D)). Single L594A and L599A mutations were introduced and assessed for their effects on viral replication. The L594A had little to no impact on viral growth, whereas L599A caused a marked replication defect that was only slightly less severe than that of the 5A-mut (Fig. 5(B) and (C)).

To confirm the impact of the NES mutation on pUL77 localization during infection, we introduced the L599A mutation into the mScarlet-tagged pUL77 HCMV reporter virus, named HCMV_dpp65_ pUL77 L599A-mScarlet (L599A-mScarlet, Fig. S5A). Under these conditions, nuclear pUL77 puncta increased from ∼3% in WT-mScarlet infected cells to ∼27% in L599A-mScarlet infected cells, resembling the redistribution observed following KPT330 treatment (Fig. S5B–C). These results indicate that proper subcellular distribution of pUL77 is essential for HCMV replication and that L599 is a key determinant of XPO1 recognition of the pUL77 NES.

We then tested whether the NES mutation altered drug sensitivity. The L599A virus displayed substantially reduced sensitivity to KPT330 compared with the WT control, consistent with impaired XPO1 engagement at this site (Fig. 5(D)). In addition, TEM analysis of L599A-infected cells revealed nuclear accumulation of B-capsids with a marked reduction in C-capsids in the absence of KPT330, closely matching the capsid maturation defect phenotype observed under XPO1 inhibition (Fig. 5(E)).

Collectively, these results demonstrate that an intact pUL77 NES, and specifically L599, is critical for XPO1-dependent nuclear distribution of pUL77 and for its role in viral genome encapsidation.

### 2.7 XPO1 inhibition blocks cytoplasmic secondary envelopment

During TEM analysis of KPT330-treated HCMV infected cells, we also observed defects in secondary envelopment within the cytoplasm. DMSO-treated cells contained abundant fully enveloped particles, whereas KPT330-treated cells accumulated unenveloped nucleocapsids surrounded by irregular vesicles. Quantitative analysis confirmed this defect, showing that unenveloped particles accounted for 72% of total cytoplasmic viral particles in KPT330-treated cells, compared with only 7% in the control group (Fig. 6(A)).

**Fig. 6:**
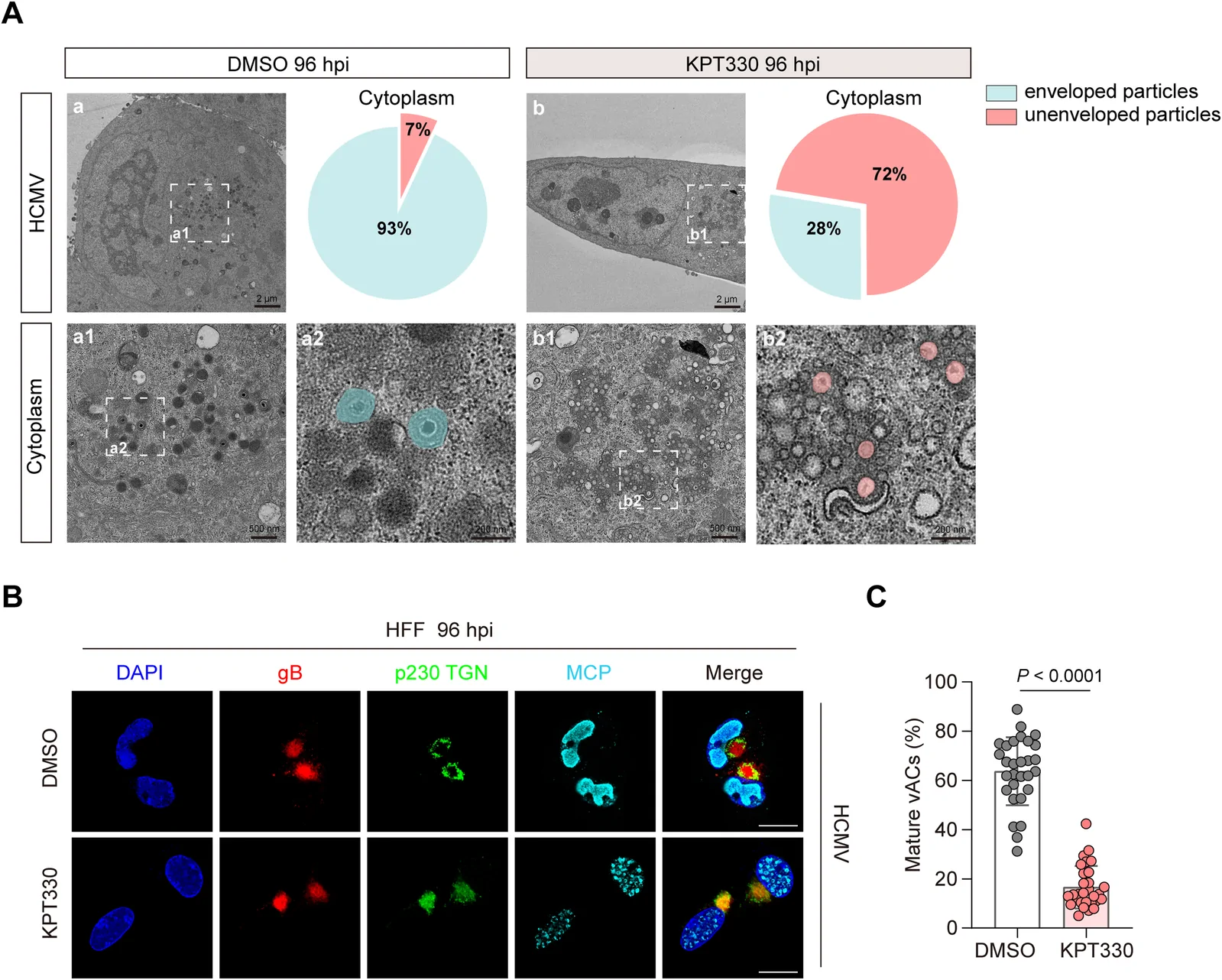
**XPO1 is required for vAC formation and viral particle envelopment.** HFFs were infected with HCMV and treated with KPT330 (0.02 μM). Cells were harvested at 96 hpi for TEM analysis or fixed at 96 hpi for IFA analysis. **(A)** Unenveloped viral particles increase in the cytoplasm. The white dashed boxes in a1 and b1 are sequentially magnified. Enveloped viral particles (including virions) are indicated in cyan, and unenveloped particles (different types of capsids) in pink. The percentages of enveloped and unenveloped particles were quantified from cytoplasmic particles in DMSO-treated (*n* = 489) and KPT330-treated cells (*n* = 434). Scale bars, 500 nm or 200 nm. **(B)** XPO1 inhibition disrupts vAC formation. The trans-Golgi network (TGN) marker p230 (green), viral glycoprotein B (gB, red), and MCP (cyan) were detected. Nuclei were counterstained with DAPI (blue). Representative images show the characteristic ring-like organization of the vAC in control cells and its fragmentation/dispersal upon XPO1 inhibition. Scale bars, 20 μm. **(C)** Quantification of vAC formation. The percentage of infected cells exhibiting mature, well-organized vACs was assessed at 96 hpi (*n* > 300 cells across 29 representative fields per condition). Data represent the mean ± SD from three independent experiments. Statistical significance was determined using a Mann–Whitney test.

Since secondary envelopment occurs within a specialized, juxtanuclear structure known as the vAC, we then examined whether XPO1 inhibition disrupts vAC formation. A mature vAC is characterized by a distinctive ring-like arrangement of the trans-Golgi network (TGN) surrounding a core where nucleocapsids undergo envelopment (Li et al., 2024). We therefore performed immunofluorescence analysis using the TGN marker p230 and viral glycoprotein B (gB) to assess vAC formation at 96 hpi. In DMSO-treated controls, 64% of infected cells displayed the classic mature vAC phenotype. In contrast, only 17% of KPT330-treated cells achieved vAC maturation; the majority remained in an immature state, characterized by the presence of gB but failure of the TGN to reorganize into the characteristic ring structure (Fig. 6(B) and (C)). These results demonstrate that XPO1 plays a critical role in the spatial organization of the vAC and the secondary envelopment of cytoplasmic viral particles.

### 2.8 XPO1 contributes to maintaining late autophagic flux required for secondary envelopment

Recent studies indicate that virus hijacks the autophagic machinery to facilitate cytoplasmic envelopment, utilizing autophagosome-like vesicles as membrane sources for secondary envelopment (Choi et al., 2018; Qin et al., 2026; Taisne et al., 2019). We therefore asked whether the cytoplasmic virion maturation defects observed following XPO1 inhibition were caused by a disruption in autophagic flux.

We first examined several well-established markers of autophagy by immunoblotting. Following treatment with KPT330, we observed pronounced accumulation of SQSTM1/p62, a selective autophagy receptor and marker of autophagic flux, along with increased levels of LC3-II, the lipidated form of the autophagosomal marker LC3 (Fig. 7(A)). In contrast, levels of ATG5, which is essential for phagophore elongation, were reduced. This pattern suggests a substantial block in autophagic flux downstream of autophagosome formation. To examine whether lysosomal degradation is impaired, we performed an LC3-II turnover assay using Bafilomycin A1 (BafA1), a V-ATPase inhibitor that prevents lysosomal acidification and thereby blocks autophagic cargo degradation. Although BafA1 alone significantly increased LC3-II levels, co-treatment with KPT330 did not further increase LC3-II levels compared with BafA1 alone (Fig. 7(B)), suggesting that KPT330 and BafA1 likely act at the same stage.

**Fig. 7:**
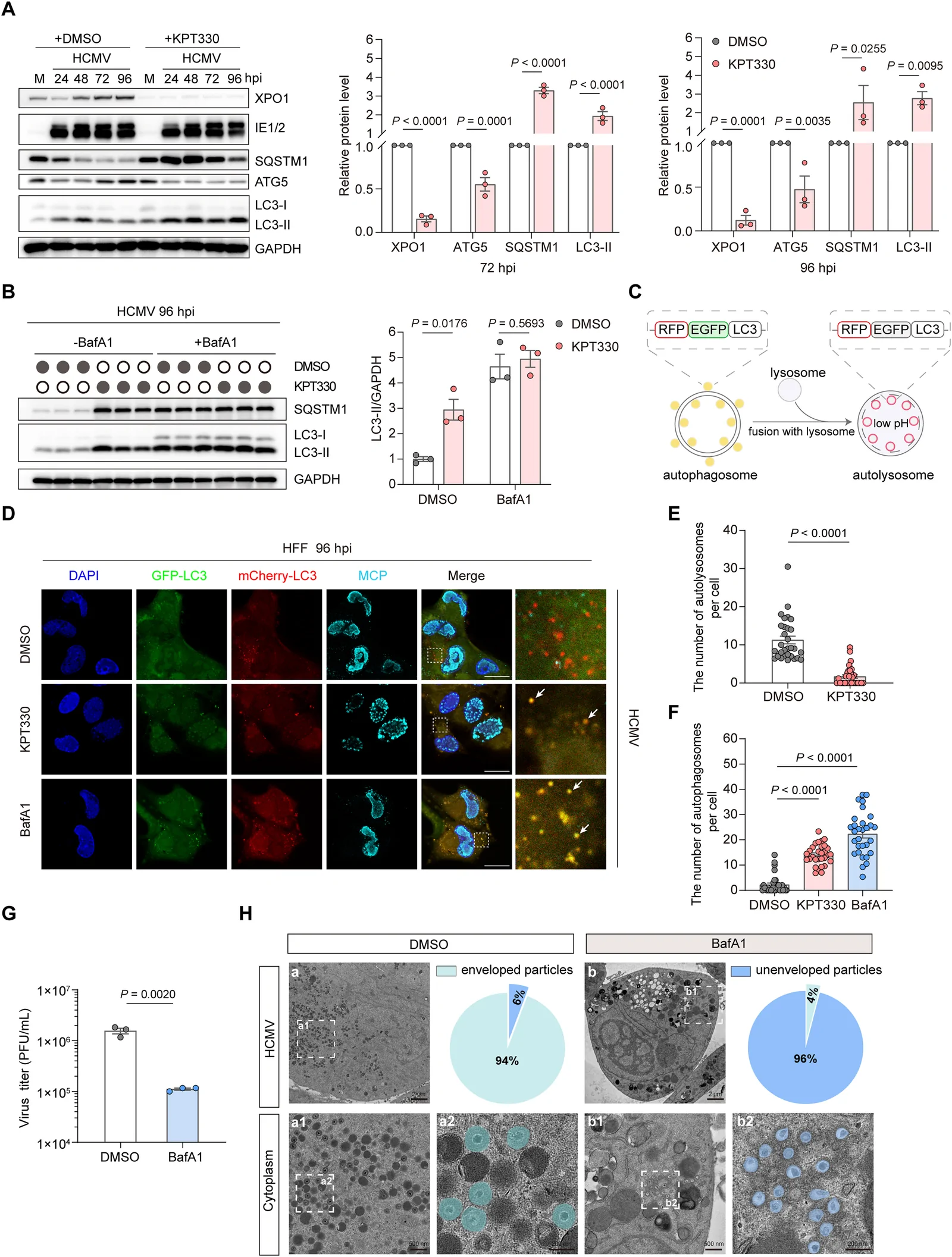
**XPO1 contributes to secondary envelopment via maintaining late autophagic flux.** **(A)** Kinetics of autophagy markers during XPO1 inhibition. HFFs were infected with HCMV (MOI = 1), treated with KPT330 or DMSO, and harvested at the indicated times post-infection. Protein levels of XPO1, ATG5, SQSTM1/p62, and LC3-II were analyzed by immunoblotting (left panel). Quantification of protein levels, normalized to GAPDH and expressed relative to DMSO-treated controls, is shown (right panel). **(B)** Assessment of autophagic flux in the presence of bafilomycin A1(BafA1). HFFs were treated with DMSO or KPT330 upon infection. BafA1 (20 nM) was added at 48 hpi to block lysosomal degradation. At 96 hpi, LC3-II levels were assessed by immunoblotting (left panel). LC3-II/GAPDH ratios were quantified using ImageJ and presented relative to the mean of the DMSO-only control (right panel). **(C)** Schematic for monitoring autophagic flux with the RFP (mCherry)-GFP-LC3 reporter. **(D)** XPO1 blockade inhibits autophagic flux. Stable RFP (mCherry)-GFP-LC3 HFF cell lines were infected with HCMV and treated with DMSO or KPT330. BafA1 (20 nM) was added at 48 hpi, and treatment lasted for 48 h. Cells were fixed at 96 hpi and stained for MCP (cyan) and DAPI (blue). The white dashed boxes are enlarged in the right panel, with white arrows indicating autophagosomes. Scale bars, 20 μm. **(E–F)** Quantification of autolysosomes and autophagosomes. The number of autolysosomes (red puncta) (E) and autophagosomes (yellow puncta) (F) per cell was quantified from panel (D). At least 150 cells for (E) and 100 cells for (F) were analyzed across 30 fields per condition. **(G)** Impact of autophagy blockade on viral progeny production. HFFs were infected with HCMV, and BafA1 (20 nM) or DMSO was added at 48 hpi. Viral titers in supernatants were determined at 96 hpi via plaque assay. **(H)** BafA1 treatment results in accumulation of unenveloped immature particles. HCMV-infected HFFs were treated with BafA1 (20 nM) or DMSO at 48 hpi and harvested at 96 hpi for TEM analysis. The boxed regions in a1 and b1 are enlarged, respectively. Enveloped particles are indicated in cyan, and unenveloped particles in blue. The percentages of enveloped and unenveloped particles were quantified from cytoplasmic particles in DMSO-treated (*n* = 1 943) and BafA1-treated cells (*n* = 1 671). Scale bars (500 nm or 200 nm) are indicated in the images. Data were collected from three independent experiments, and results are presented as mean ± SEM. Statistical significance was assessed using two-way ANOVA (A, B), the Mann–Whitney test (E), the Kruskal–Wallis test (F), or a two-tailed unpaired *t*-test (G).

To visualize the effects of XPO1 inhibition on autophagic flux, we evaluated autophagosome and autolysosome formation in HFFs stably expressing the RFP (mCherry)-EGFP-LC3 reporter. In the acidic environment of mature autolysosomes, the GFP signal is quenched while the RFP signal persists; thus, yellow puncta (GFP+/RFP+) represent autophagosomes, whereas red-only puncta (GFP−/RFP+) represent autolysosomes (Fig. 7(C)). XPO1 inhibition resulted in a marked increase in yellow autophagosomes and a significant decrease in red autolysosomes (Fig. 7(D–F)). This shift mirrored the phenotype induced by BafA1, strongly suggesting that XPO1 inhibition blocks the transition from autophagosomes to degradative autolysosomes.

Finally, we tested whether this late-stage disruption of autophagic flux is sufficient to cause secondary envelopment defects. We treated HCMV-infected cells with BafA1 at 48 hpi and measured viral yields at 96 hpi. BafA1 treatment significantly reduced viral titers (Fig. 7(G)). Further TEM analysis showed that only 4% of cytoplasmic viral particles were fully enveloped in BafA1-treated cells, which is dramatically lower than the 94% observed in control cells (Fig. 7(H)). These results further confirm that disruption of late-stage autophagic flux impairs viral envelopment in the cytoplasm.

Together, these findings demonstrate that XPO1-dependent regulation of late-stage autophagic flux contributes to efficient secondary envelopment of HCMV in the cytoplasm.

## 3 Discussion

HCMV maturation is a complex, multi-step process that depends on tight coordination of nuclear and cytoplasmic events. In this study, we identify XPO1 as a key host factor involved in both nuclear viral genome encapsidation and cytoplasmic secondary envelopment during HCMV maturation. XPO1 regulates the proper nuclear localization of pUL77, which is crucial for efficient viral genome packaging. Disruption of XPO1 function leads to aberrant nuclear distribution of pUL77, defective capsid maturation, and impaired viral replication. Furthermore, we demonstrate that XPO1 is required for vAC formation and maintenance of late-stage autophagic flux, which is necessary for cytoplasmic envelopment.

As a core component of the encapsidation machinery, pUL77 interacts directly with the terminase subunits pUL56 and pUL89, as well as the CVSC protein pUL93 (Borst et al., 2016; Köppen-Rung et al., 2016). Loss or functional impairment of pUL77 leads to accumulation of B-capsids, underscoring its essential role in encapsidation (Borst et al., 2016; Gourin et al., 2025). Structural studies have shown that pUL77 oligomers are located at or near the portal vertex, where they cap the packaged genome and seal the capsid (Li et al., 2021, 2023). These studies provide important structural insights into encapsidation, yet the spatial regulation of pUL77 has remained poorly defined. HCMV pUL77 was characterized as a capsid maturation factor that functions within the nucleus, where genome packaging occurs. Accordingly, previous studies primarily focused on pUL77 NLSs, nuclear targeting, and genome packaging, rather than nuclear export (Gourin et al., 2025; Köppen-Rung et al., 2016). In addition, XPO1-dependent NESs are compositionally diverse leucine-rich motifs and may not be readily identified through approaches focused on NLS motifs or nuclear localization. Our study used a complementary strategy specifically designed to interrogate XPO1-dependent nuclear export. We combined a functional NES reporter assay with NES motif prediction to identify candidate NESs, which were further evaluated through structural modeling, targeted mutagenesis, and infection-based validation. This analysis identified a previously unrecognized XPO1-dependent NES within pUL77, providing a methodological template for NES identification in other proteins.

Notably, the L599A mutant did not recapitulate the pUL77 puncta frequency observed with pharmacological XPO1 inhibition. A plausible explanation is that the L599A mutation retains residual XPO1-binding activity, as indicated by co-immunoprecipitation experiments (Fig. S6). Moreover, because XPO1 inhibitors broadly disrupt XPO1-dependent nuclear export, other XPO1-dependent proteins may also contribute to puncta formation. Thus, our results indicate that the L599A mutation represents a partial disruption of NES-dependent pUL77 export rather than a complete phenocopy of XPO1 inhibition. On the other hand, the NES mutant (L599A) exhibited two distinct patterns: in some cells, it resembled wild-type pUL77, whereas in others it formed spherical dot-like aggregates (Fig. S5B). MCP followed these altered pUL77 distributions, indicating that changes in pUL77 localization can concentrate capsid assembly machinery at specific nuclear sites. By contrast, pUL56 and pUL93, which are also involved in DNA packaging and interact with XPO1, did not display nuclear aggregation upon XPO1 inhibition. The spherical morphology of these aggregates raises the possibility that pUL77 may possess an intrinsic propensity for liquid-liquid phase separation, similar to the behavior reported for HCMV UL112-113 proteins (Caragliano et al., 2022). However, this hypothesis requires further experimental validation. We propose a speculative model in which continuous XPO1-mediated export may act as a spatial regulatory mechanism to keep nuclear pUL77 below the threshold required for phase transition, thereby maintaining a diffuse nuclear pool to support efficient viral genome encapsidation.

Our identification of pUL77 establishes it as the third HCMV protein shown to date to depend on XPO1-mediated nuclear export, following pUL94 and pp65 (Liu et al., 2012; Sanchez et al., 2007). Meanwhile, XPO1 inhibition induced pp65 to form nuclear dense speckles, which were not observed in the previous study (Sanchez et al., 2007). The discrepancy is likely due to limited antibody access, as aggregates were absent with anti-pp65 staining but clearly visible with a pp65-mScarlet fusion (Fig. S2B). TEM analysis using a pp65 deletion strain further confirmed that the nuclear dense speckles correspond to pp65-containing structures. pp65 is a self-associating protein that can form nuclear inclusions in uninfected cells, similar to our observations (Cui et al., 2009), and pUL97 kinase activity is required to prevent this nuclear aggregation (Prichard et al., 2005). Thus, pUL97-mediated phosphorylation of pp65 probably activates its nuclear export signal and prevents nuclear accumulation. Nevertheless, we did not explore this further, as pp65 deletion did not rescue the capsid defects caused by XPO1 inhibition, indicating that the nuclear dense speckles do not sequester essential components of the capsid assembly machinery.

This study also implicates XPO1 in autophagy regulation. Current studies indicate that XPO1 inhibition can either enhance or suppress autophagy depending on context. In *C. elegans* and glioblastoma cells, XPO1 blockade traps the transcription factor EB in the nucleus, upregulates autophagy-related genes, and promotes autophagic flux (Silvestrini et al., 2018; Tang et al., 2025). In contrast, during starvation-induced autophagy and in certain olaparib-treated glioblastoma settings, XPO1 inhibition has been shown to impair autophagic flux and stall progression at later stages of autophagosome maturation or lysosomal degradation (Martin et al., 2019; Wang et al., 2024), similar to the autophagy-related defects we observe in HCMV-infected HFFs. In Kaposi's sarcoma-associated herpesvirus (KSHV)-infected cells, XPO1 inhibition causes nuclear accumulation of the autophagy adaptor p62, yet does not alter LC3-II processing or p62 turnover, indicating that autophagic flux is not impaired; instead, nuclear p62 enhances TBK1-IRF3 signaling and antiviral responses (Meng & Gao, 2021). These observations indicate that how XPO1 regulates autophagy depends on the host signaling network and specific virus-host interactions. For HCMV in the late phase of replication, intact XPO1 function appears to be critical for maintaining efficient autophagosome maturation.

The role of autophagy in HCMV infection is also highly context-dependent. Early studies showed that autophagy induction reduces HCMV infection, whereas its inhibition enhances viral replication and virion release (Belzile et al., 2016; Clark et al., 2018; Zimmermann et al., 2021), indicating that active autophagy restricts HCMV. However, viral manipulation of autophagy is dynamic: HCMV initially stimulates autophagic flux and autophagosome formation, but then rapidly targets Beclin 1, mTORC1, or the TSC1/2 complex to suppress autophagy (Chaumorcel et al., 2008, 2012; Kudchodkar et al., 2004; Moorman et al., 2008). Previous studies have reached divergent conclusions regarding the role of autophagy in HCMV maturation. Zimmermann et al. found that inhibition of LC3 lipidation did not impair secondary envelopment, whereas Taisne et al. reported that perturbation of multiple autophagy-related components reduced HCMV production and suggested a role for the autophagy machinery in final cytoplasmic envelopment (Taisne et al., 2019; Zimmermann et al., 2021). These differences may reflect the distinct approaches used to perturb autophagy and the specific stages of the pathway affected. Importantly, despite differences in their overall conclusions, these studies share several observations relevant to HCMV maturation. In particular, autophagy-related components such as p62 and LC3-II localize to the vAC and enveloped virions, suggesting an association between autophagy-related membranes and HCMV maturation.

A recent study further showed that HCMV suppresses lysosomal acidification and autophagic cargo degradation by US33A-mediated degradation of DMXL1, yet vAC formation still requires lysosomes, and US33A expression delays vAC biogenesis (Li et al., 2024). Consistent with this model, XPO1 inhibition reduces lysosomal acidification (Fig. S7), stalls autophagosome maturation, disrupts vAC formation, and is associated with defective secondary envelopment, closely mimicking the phenotype induced by BafA1. However, how impaired autophagic/lysosomal function contributes to the envelopment defect requires further investigation. Collectively, these findings indicate that HCMV relies on finely tuned autophagosome–lysosome dynamics, rather than on global autophagy induction or blockade during the late stages of replication, and that XPO1-dependent autophagosome maturation contributes to HCMV secondary envelopment.

Our findings point to a promising new direction for antiviral development. Because XPO1 is a host factor required for multiple steps in HCMV maturation, targeting it is likely to impose a higher barrier to resistance than current viral-targeting antivirals. In addition, any mutation in pUL77 that reduces susceptibility to XPO1 inhibition would likely also impair its nuclear export, further constraining the virus's ability to evolve effective escape routes, especially under prolonged or prophylactic treatment. Importantly, XPO1 inhibitors such as Selinexor (KPT330) are already FDA-approved oral drugs for relapsed or refractory multiple myeloma and certain lymphomas, with additional XPO1-targeted regimens in active clinical trials for both hematologic and solid tumors (Chen & Adams, 2025; Chirino et al., 2024). Repurposing these agents for HCMV could dramatically accelerate the path from bench to bedside. XPO1 inhibition has also been shown to suppress replication of diverse viruses, including influenza A and B, HIV-1, KSHV, and respiratory syncytial virus (Mathew et al., 2021; Meng & Gao, 2021; Mghezzi-Habellah et al., 2023). Nevertheless, given the essential role of XPO1 in cellular nuclear export, pharmacological inhibition may cause undesirable side effects. Clinical use of the XPO1 inhibitor selinexor in hematological malignancies has been associated with adverse events, including neutropenia, thrombocytopenia, weight loss, and fatigue (Chari et al., 2019; Gavriatopoulou et al., 2020). These adverse events are generally dose-dependent and can be managed through dose optimization and supportive care (Azmi et al., 2021). Thus, repurposing clinically available XPO1 inhibitors, together with the development of more selective next-generation compounds, may provide a promising host-directed, broad-spectrum antiviral strategy against XPO1-dependent viruses.

In conclusion, we identify XPO1 as a crucial host determinant that coordinates nuclear genome encapsidation and cytoplasmic envelopment during HCMV infection. By regulating the nuclear export of pUL77, XPO1 ensures proper capsid maturation, and by facilitating autophagosome maturation, it supports efficient cytoplasmic envelopment. These findings broaden our understanding of HCMV maturation and suggest that targeting XPO1 could be an effective strategy for developing host-directed antivirals.

## 4 Materials and methods

### 4.1 Ethics statement

Human foreskin fibroblasts (HFFs) were isolated and maintained in the laboratory as described previously (Yang et al., 2021; Zhou et al., 2026). The cell isolation was approved by the Institutional Review Board (IRB) of the Wuhan Institute of Virology, Chinese Academy of Sciences (Approval number WIVH10201202), in accordance with the Guidelines for Biomedical Research Involving Human Subjects. The tissue was obtained from a postmortem fetus and written informed consent was waived.

All experiments involving infectious virus were reviewed and approved by the Institutional Biosafety Committee (IBC) and conducted in Biosafety Level 2 (BSL-2) laboratory facilities in compliance with institutional standard operating procedures and biosafety regulations.

### 4.2 Cells and cell culture

HFFs were cultured in minimum essential medium (MEM, Gibco, 41500034) supplemented with 10% fetal bovine serum (FBS; Gibco, 10099141C) and penicillin-streptomycin (100 U/mL and 100 μg/mL, respectively; Gibco, 15140122). Human embryonic kidney 293T cells (CRL–11268) were purchased from the American Type Culture Collection. HEK293T cells were cultured in Dulbecco's modified Eagle medium (DMEM, Gibco, 11995123) supplemented with 10% FBS and penicillin-streptomycin as described above. All cell cultures were maintained at 37 °C in a humidified atmosphere containing 5% CO_2_.

### 4.3 Viruses, recombinant variants, and infection

The HCMV Towne strain (HCMV, ATCC VR-977) was propagated and titrated using a plaque assay in HFFs. Recombinant viruses were derived from the HCMV Towne BAC (bacterial artificial chromosome, BAC), following published protocols (Tischer et al., 2010). The HCMV_dpp65_ was generated by replacing the pp65 gene with an ampicillin resistance cassette, and HCMV_dpp65_ pUL77-mScarlet was generated by inserting the red fluorescent protein mScarlet at amino acid residue 154 of pUL77, based on a previously reported study (Borst et al., 2016). HCMV Myc-pUL56 was generated by incorporating a Myc tag at the N-terminus of pUL56.

A series of pUL77 NES mutations (5A-mut, L594A, and L599A) were introduced into an EGFP-expressing HCMV_dpp65_ BAC using the markerless “en passant” mutagenesis methodology. In addition, the pUL77 L599A mutation was also incorporated into the backbone of HCMV_dpp65_ pUL77-mScarlet to generate the corresponding recombinant virus. BAC isolation from bacterial cultures was performed using the NucleoBond Xtra Midi kit (Macherey-Nagel, 740410.50) according to the manufacturer's instructions. Successful mutagenesis of each BAC was confirmed by restriction analysis and sequencing. To reconstitute virus progeny from these HCMV BAC genomes, the BACs were transfected into HFFs, and recombinant mutant viruses were collected when the entire monolayer of cells was lysed. HCMV infection was performed at an MOI of 1, and samples were harvested at the time points indicated in the figures.

### 4.4 Plaque assays

HFFs were seeded into 24-well plates at a density of 1.5 × 10^6^ cells per plate. On the following day, 200 μL of 10-fold serially diluted supernatant samples was added to the cell monolayers and adsorbed for 3 h at 37 °C. Each well was overlaid with MEM containing 0.5% agarose. Plaques were counted at 7–10 days post incubation, and the average titer was calculated from three independent experiments.

### 4.5 Plasmids, shRNAs and transfection

Plasmid construction was described previously (Zhou et al., 2026). In brief, UL51, UL56, UL77, UL89, and UL93 fragments were amplified from the HCMV_BAC_ genome and cloned into the 3×Flag vector using the ClonExpress II One Step Cloning kit (Vazyme, C112). UL77-mScarlet was amplified from the HCMV_dpp65_ pUL77-mScarlet genome and inserted into the pCDH vector. UL89, UL93, and HIV Rev fragments were fused to mScarlet by PCR amplification and cloned into the pCDH vector by homologous recombination. The HIV-Rev-mScarlet plasmid was digested with *Eco*RI (Takara, 1040A), and six pUL77 fragments were subsequently inserted into the linearized vector.

The shRNAs targeting XPO1 were synthesized by Sangon Biotech, and the sequences used are listed in Table S2. The shScram, shXPO1-1, and shXPO1-2 were cloned into the pCDH vector, and all constructs were confirmed by DNA sequencing. Lentiviruses were produced in HEK293T cells as previously described (Zhou et al., 2026). Briefly, the lentiviral plasmids pMD2.G and psPAX2, and the target transfer plasmid were co-transfected into 70%–80% confluent HEK293T cells at a 1:1:1 ratio. The supernatant containing lentivirus was harvested at 48 h and 72 h post-transfection and stored at −80 °C.

Plasmid or HCMV BAC transfections were performed using Neofect™ DNA transfection reagent (NEOFECT, TF201201) according to the manufacturer's instructions. For 12-well plates, 1 μg of DNA was mixed with 1 μL of transfection reagent in 50 μL of Opti-MEM (Gibco, 41500034) and added to cells at 50%–70% confluence. For other culture plates, DNA and reagent amounts were scaled proportionally to the well surface area. Cells were maintained under standard culture conditions as described above.

### 4.6 Cell viability test

The 3-(4,5-dimethylthiazol-2-yl)-2,5-diphenyltetrazolium bromide (MTT) assay (Sigma-Aldrich, M2128) was used to evaluate the cytotoxicity of KPT330 (MCE, HY-17536) and KPT335 (MCE, HY-15970) in HFFs according to the manufacturer's instructions. Briefly, cells seeded in 96-well plates were incubated with increasing concentrations of the drugs. After 96 h of incubation at 37 °C, the culture medium was replaced with 200 μL of fresh phenol red-free MEM containing 0.5% MTT. Following an additional 4 h incubation, the medium was removed, and 150 μL of dimethyl sulfoxide (DMSO) was added to each well. Absorbance was then measured at 570 nm.

### 4.7 Immunofluorescence analysis (IFA)

Synchronized cells were reseeded onto coverslips and infected with virus after attachment. After infection, coverslips were fixed with 4% paraformaldehyde at the indicated post-infection time points, and target proteins were detected by incubation with primary antibodies followed by the appropriate secondary antibodies, as previously described (Zhou et al., 2026). Nuclei were counterstained with DAPI (4′,6-diamidino-2-phenylindole, Sigma-Aldrich, D9542). Images were acquired using either a Leica STELLARIS 8 FALCON DLS laser confocal microscope (LAS X software) or an Andor confocal microscope (Dragonfly 202).

### 4.8 Immunoblotting and co-immunoprecipitation (Co-IP)

Cells were harvested and lysed in cell lysis buffer (Beyotime, P0013) supplemented with a protease inhibitor cocktail (Roche, 04693159001). Protein concentrations of the lysates were quantified using the Bradford assay (Bio-Rad, 500–0205). Equal amounts of protein from each lysate were separated by SDS–polyacrylamide gel electrophoresis (SDS-PAGE) and transferred onto polyvinylidene difluoride (PVDF) membranes (Millipore, ISEQ00010). PVDF membranes were incubated overnight at 4 °C with primary antibodies, followed by a 1-h incubation at room temperature with horseradish peroxidase–conjugated secondary antibodies (Table S3). Protein detection was performed using SuperPico ECL Chemiluminescence Kit (Vazyme, E422–02), and signals were visualized with a FluorChem HD2 imaging system (Alpha Innotech). Protein levels were quantified by densitometric analysis using ImageJ software (National Institutes of Health, USA).

For immunoprecipitation assays, cell lysates were incubated with protein G agarose and either control IgG or the appropriate antibodies. The precipitants were then washed five times with lysis buffer and subjected to immunoblotting analysis.

### 4.9 Sequence alignment

Multiple sequence alignments were performed using Clustal Omega provided by the EMBL-EBI bioinformatics web tools. Amino acid conservation was visualized using Jalview.

### 4.10 Structure modeling

The interaction between XPO1 and the pUL77-NES was modeled from sequence information using AlphaFold3, which was run through its public web server (https://www.alphafoldserver.com). AlphaFold3 generated five models for each complex, which were ranked according to the model ranking score (Table S4) (Abramson et al., 2024). Structural visualization and analysis were performed using ChimeraX 1.8.

### 4.11 Transmission electron microscopy (TEM)

Cells were infected with HCMV (MOI = 1, 96 hpi), fixed in 2.5% (wt/vol) glutaraldehyde for 1 h at room temperature, and the samples were postfixed in 1% osmium tetroxide. The specimens were then dehydrated through a graded series of ethanol concentrations (30% to 100%) and embedded using an Embed 812 kit (Electron Microscopy Sciences, Fort Washington, PA). Ultrathin sections (60–80 nm) of the embedded specimens were prepared and placed onto Formvar-coated copper grids (200 mesh), stained with 2% (wt/vol) phosphotungstic acid (PTA, pH 6.8), and examined with a Tecnai transmission electron microscope (FEI).

### 4.12 Inhibitor treatments

KPT330 (MCE, HY–17536) and KPT335 (MCE, HY–15970) were dissolved in DMSO. For determination of IC_50_ and CC_50_, a 100 mM stock solution was serially diluted to 100, 31.6, 10, 3.16, 1, 0.316, 0.1, 0.031 6, 0.01, and 0.001 μM. In infection experiments, 0.02 μM KPT330 or KPT335 was maintained throughout infection until harvest, with DMSO used as the vehicle control. Bafilomycin A1 (BafA1, MCE, HY–100558) was added to a final concentration of 20 nM at 48 hpi, and cultures were incubated for an additional 48 h before sample collection.

### 4.13 Statistical analysis

Statistical analyses were conducted using GraphPad Prism 8.4.3. Statistical significance was calculated by the chi-square test, unpaired *t*-test, Mann–Whitney test, Kruskal–Wallis test, one-way ANOVA with Dunnett's T3 multiple-comparisons test, or two-way ANOVA, as indicated in the figure legends. No data points have been excluded from the analyses.

## CRediT authorship contribution statement

**Jin-Peng Wu:** Writing – original draft, Visualization, Validation, Software, Resources, Methodology, Investigation, Formal analysis, Data curation, Conceptualization. **Yue-Peng Zhou:** Methodology. **Meng-Jie Mei:** Methodology. **Yu-Ting Pan:** Methodology. **Jin-Yan Sun:** Resources, Methodology. **Wen-Bo Zeng:** Resources, Methodology, Funding acquisition. **Han Cheng:** Writing – review & editing, Writing – original draft, Supervision, Funding acquisition, Conceptualization. **Min-Hua Luo:** Writing – review & editing, Supervision, Resources, Funding acquisition, Conceptualization. **Bo Yang:** Writing – review & editing, Writing – original draft, Supervision, Methodology, Investigation, Funding acquisition, Conceptualization.

## Data and materials availability

All the data are available upon request from the corresponding authors. The plasmids and virus are available from the corresponding authors upon request.

## Funding

This work was supported by grants from National Key R&D Program of China (2023YFC2306600 to M.H.L. & W.B.Z.), National Natural Science Foundation of China (32570159 to H.C. and 32370168 to B.Y.), Strategic Priority Research Program of the Chinese Academy of Sciences (XDB1610000 to M.H.L.), Key Program of Natural Science Foundation of China (82430070 to M.H.L.), Hunan Provincial Science and Technology Innovation Program Project (2024WK2016 to M.H.L. & B.Y.).

## Declaration of competing interest

The authors declare that they have no known competing financial interests or personal relationships that could have appeared to influence the work reported in this paper.

## Figures and Tables

**Fig. S1:**
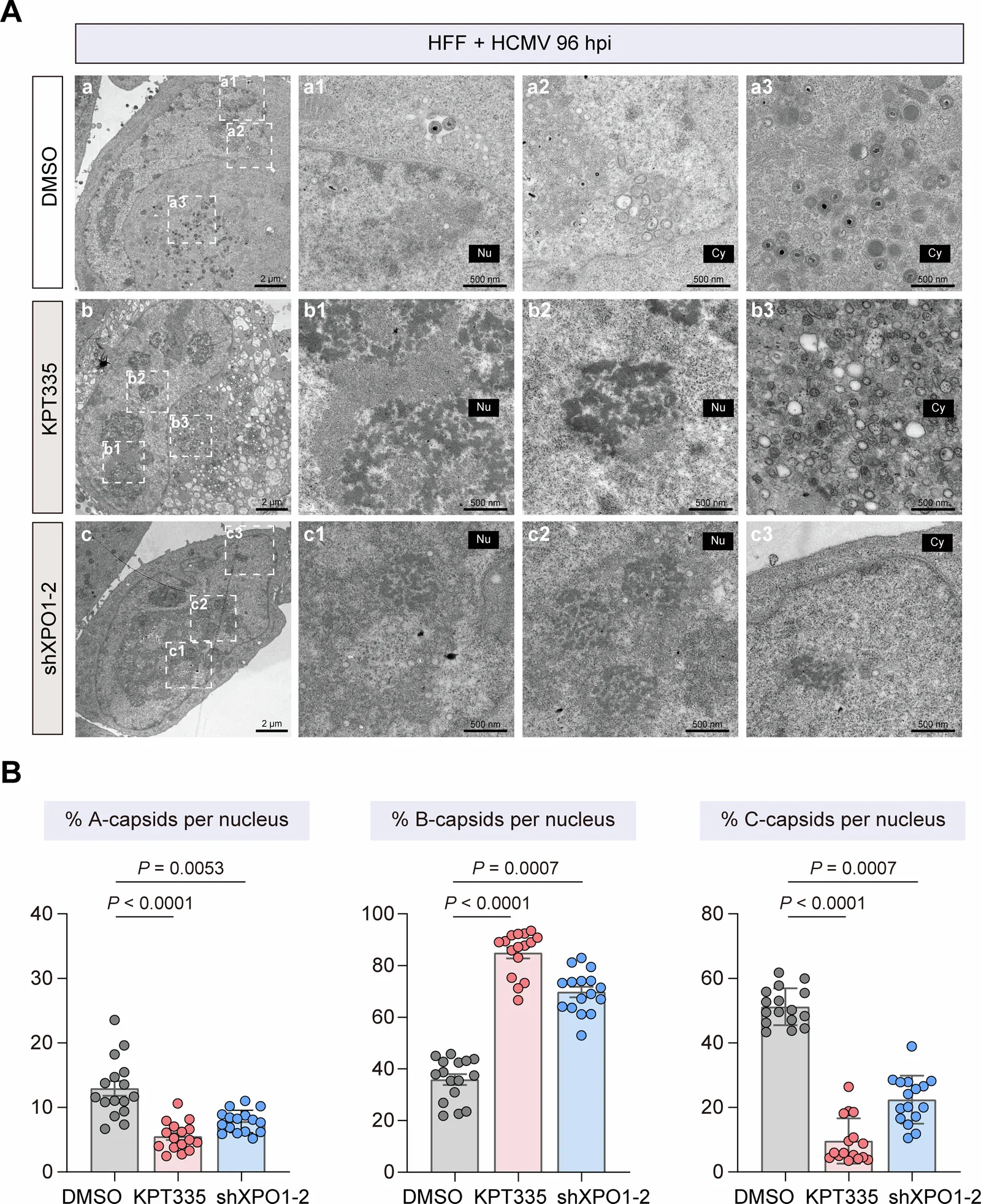
**Targeting XPO1 leads to impairment of nuclear capsid maturation and appearance of dense speckles.** **(A)** HFFs were infected with HCMV, followed by treatment with KPT335 (0.02 μM) or DMSO, whereas shXPO1-2 HFFs were infected with HCMV alone (MOI = 1). Cells were fixed at 96 hpi for TEM analysis. Scale bars (2 μm or 500 nm) are indicated in the images. Nu, nucleus; Cy, cytoplasm. **(B)** Quantification of nuclear capsid populations (*n* = 16 nuclei per condition; error bars represent mean ± SD). *P* values were calculated by the Kruskal–Wallis test followed by Dunn's multiple comparisons test.

**Fig. S2:**
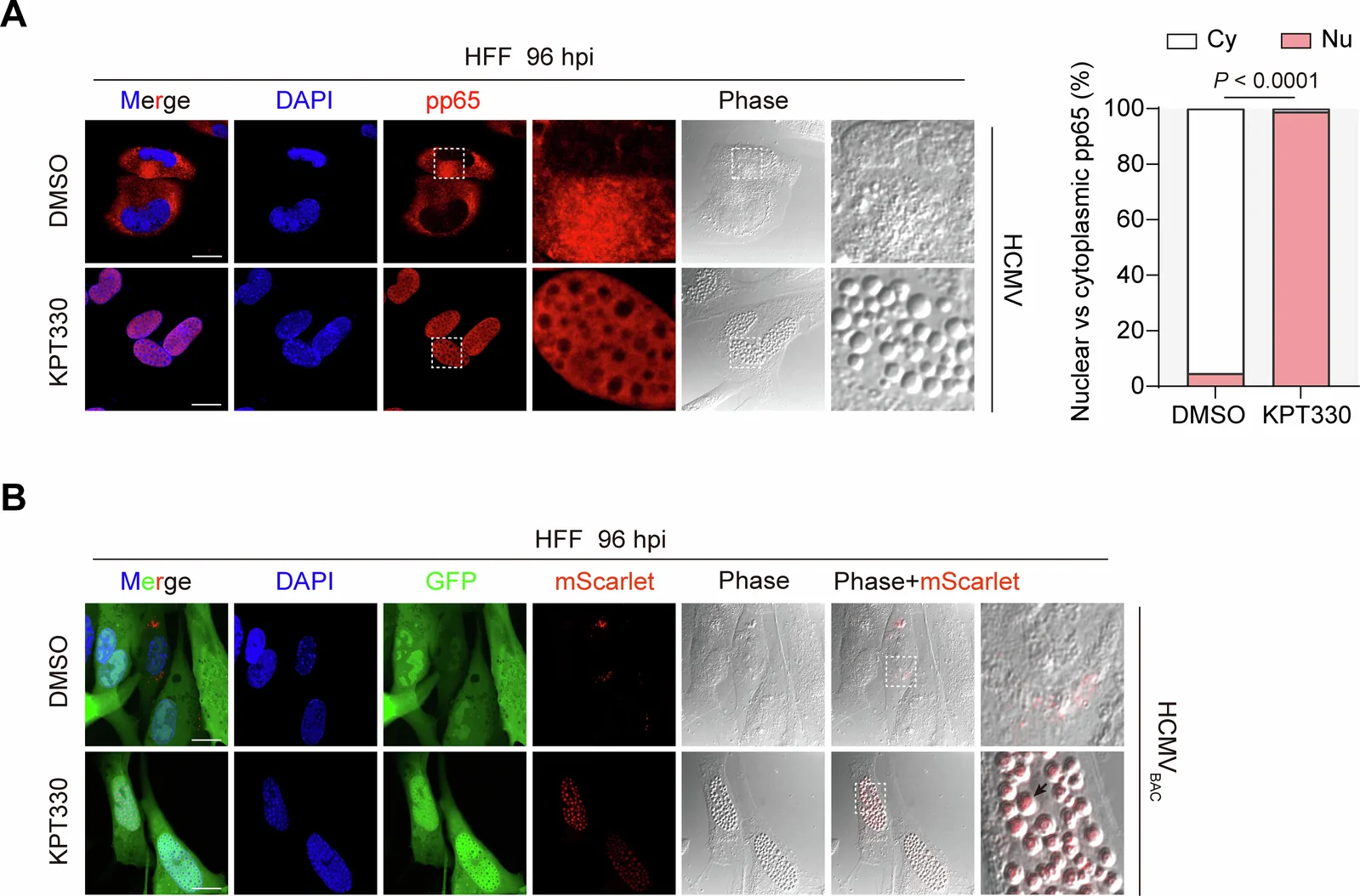
**HCMV pp65 is involved in the formation of dense spherical bodies.** **(A)** XPO1 inhibition induces nuclear retention of pp65. Infected HFFs were treated with either KPT330 or DMSO, fixed at 96 hpi, and stained for pp65 (red) and DAPI (blue). Dense spherical bodies were visualized by light microscopy. Scale bars: 20 μm (left). Quantification of pp65 nuclear–cytoplasmic distribution at 96 hpi (*n* = 280 for DMSO; *n* = 264 for KPT330, right). Nu, nucleus; Cy, cytoplasm. *P* value was calculated by the chi-square test. **(B)** HCMV pp65-mScarlet localizes to dense spherical bodies. HFFs were transfected with a plasmid expressing pp65-mScarlet. At 48 h post-transfection, cells were infected with HCMV_BAC_ and treated with KPT330 (0.02 μM). At 96 hpi, the localization of pp65-mScarlet was detected by confocal microscopy, and nuclei were counterstained with DAPI. The white dashed boxes are enlarged in the right panel, with black arrows indicating dense spherical bodies. Scale bars, 20 μm.

**Fig. S3:**
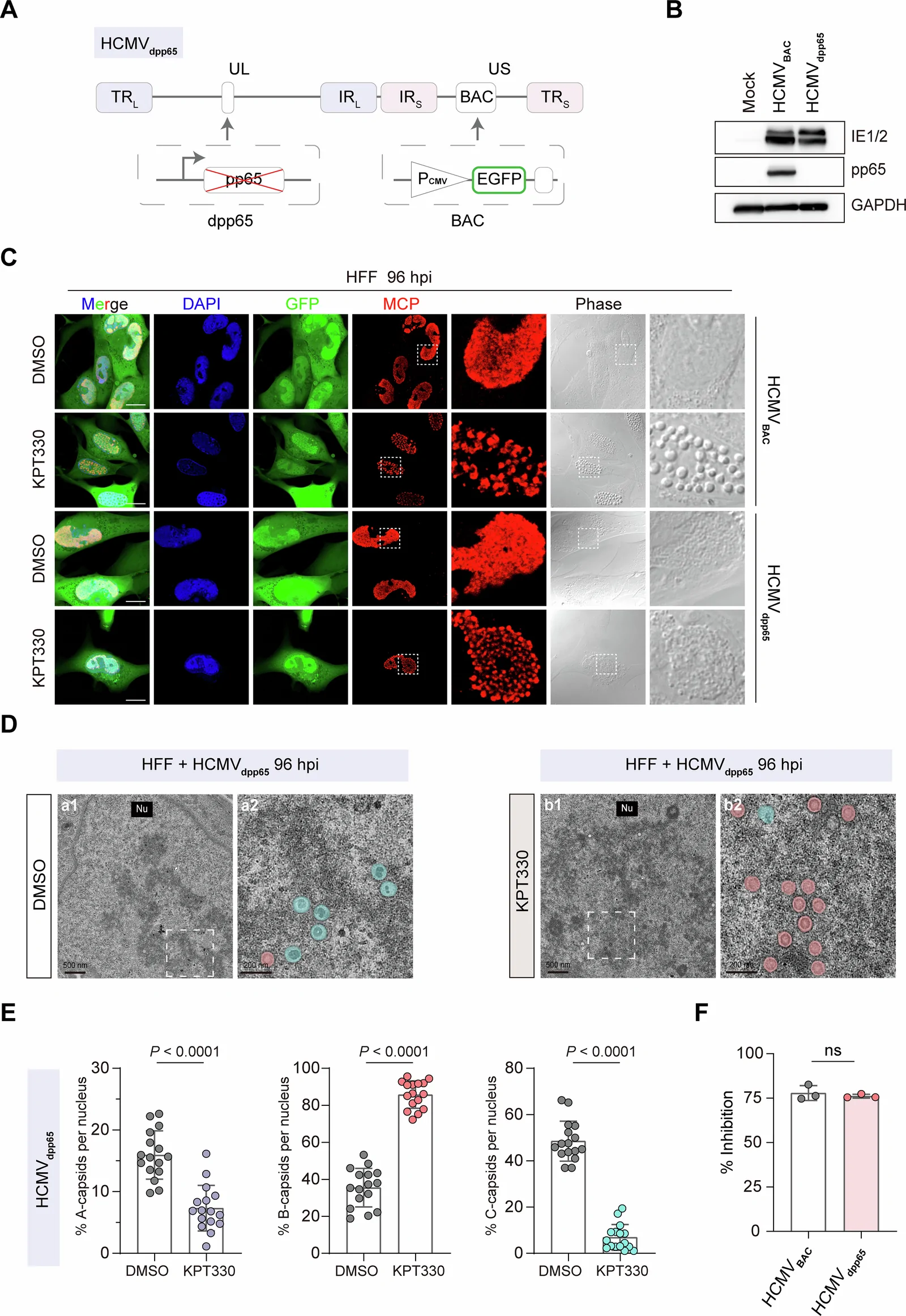
**Nuclear retention of pp65 drives dense speckle formation, independent of viral genome encapsidation defects.** **(A)** Schematic genome diagram of HCMV_dpp65_. The pp65 open reading frame was replaced by a kanamycin resistance cassette via homologous recombination. **(B)** Validation of pp65 deletion. HFFs were infected with HCMV_BAC_ or HCMV_dpp65_ (MOI = 1), fixed at 96 hpi, and analyzed by immunoblotting for IE1/2, pp65, and GAPDH (as a loading control). Mock-infected HFFs served as a negative control. **(C)** HCMV pp65 is responsible for the formation of dense spherical bodies but not for MCP redistribution. HFFs were infected with HCMV_BAC_ or HCMV_dpp65_ and treated with either KPT330 or DMSO. Cells were fixed at 96 hpi, and target localization was determined by immunofluorescence analysis (IFA). Dense spherical bodies were visualized by light microscopy. GFP (green) marks infected cells, MCP (red) indicates capsid localization, and nuclei were counterstained with DAPI (blue). The white dashed box is enlarged in the right panel. Scale bars, 20 μm. **(D)** TEM analysis of HCMV_dpp65_ assembly. HFFs infected with HCMV_dpp65_ were treated with KPT330 (0.02 μM) or DMSO and examined by TEM at 96 hpi. C-capsids are indicated in cyan, and B-capsids in pink. The white dashed box is enlarged in the right panel. Scale bars (500 nm and 200 nm) are indicated in the images. Nu, nucleus. **(E)** Quantification of capsid types in HCMV_dpp65_-infected nuclei. The proportions of A-, B-, and C-capsids were determined from 16 HCMV_dpp65_-infected nuclei in each treatment group. **(F)** Antiviral efficacy of KPT330 is independent of pp65. HFFs were infected with HCMV_BAC_ or HCMV_dpp65_ (MOI = 1) and treated with either KPT330 or DMSO. Viral titers were determined at 96 hpi by plaque assay. Results are normalized to the respective DMSO-treated controls. ns, not significant (*P* > 0.05). Data are represented as mean ± SD. *P* values were calculated by the Mann–Whitney test (E), or two-tailed unpaired *t*-test (F).

**Fig. S4:**
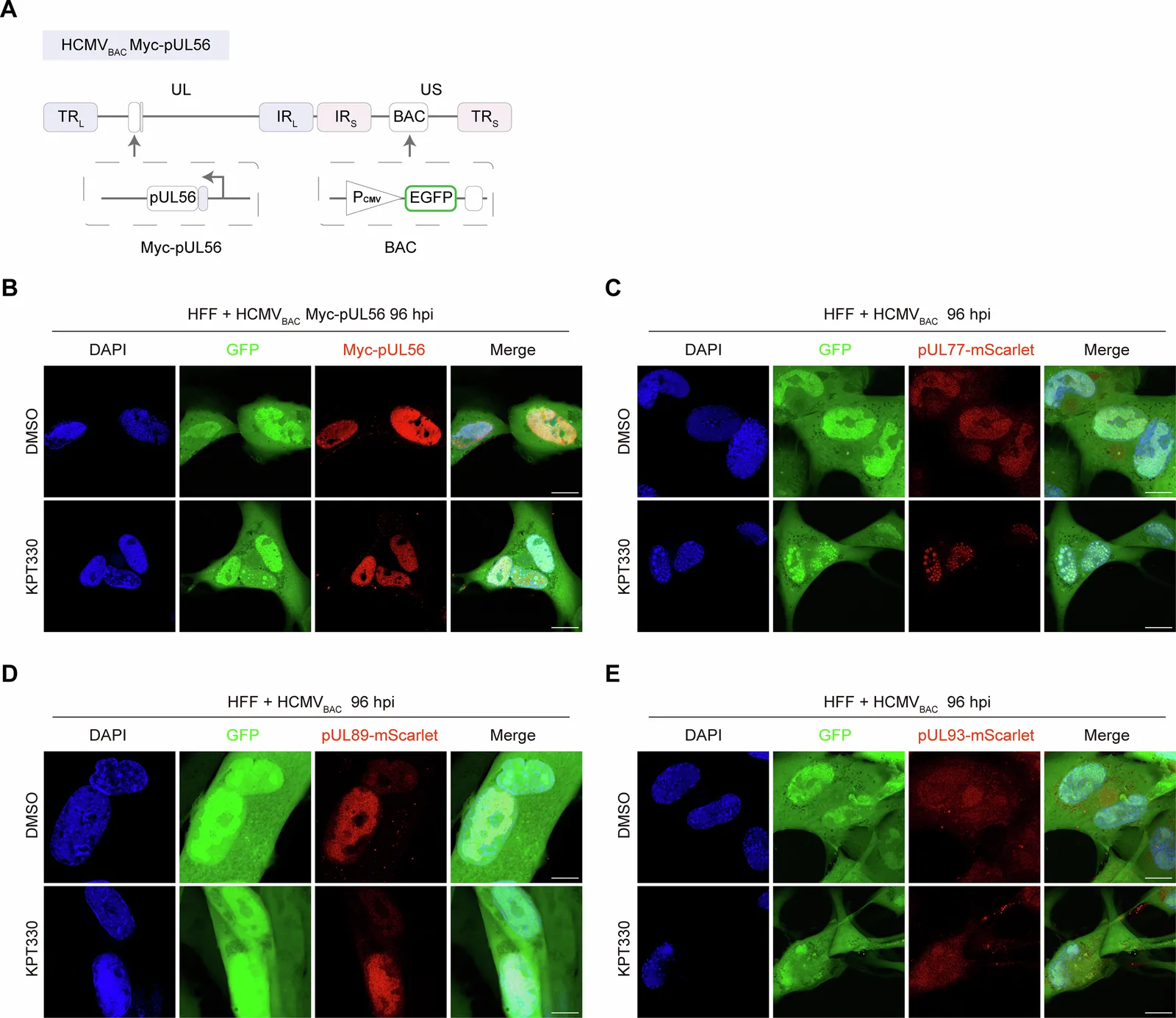
**Subcellular distribution of XPO1-interacting viral proteins upon XPO1 inhibition.** **(A)** Schematic genome diagram of HCMV_BAC_ Myc-pUL56. The Myc tag was inserted at the N terminus of pUL56 by recombination. **(B)** Localization of Myc-pUL56 in the presence of XPO1 inhibition. Representative images showing the subcellular distribution of Myc-pUL56 in recombinant HCMV_BAC_-infected cells treated with KPT330 or DMSO control. **(C-E)** Distribution of pUL77, pUL89 and pUL93 upon XPO1 inhibition. HFFs were transfected with pUL77-mScarlet (C), pUL89-mScarlet (D) or pUL93-mScarlet (E) for 48 h, followed by infection with HCMV_BAC_ (MOI = 1) and treatment with KPT330 (0.02 μM). Cells were fixed at 96 hpi, and the localization of mScarlet (red) was detected by IFA. Nuclei were counterstained with DAPI (blue). Scale bars, 10 μm (in (D)) and 20 μm (in (B), (C), (E)).

**Fig. S5:**
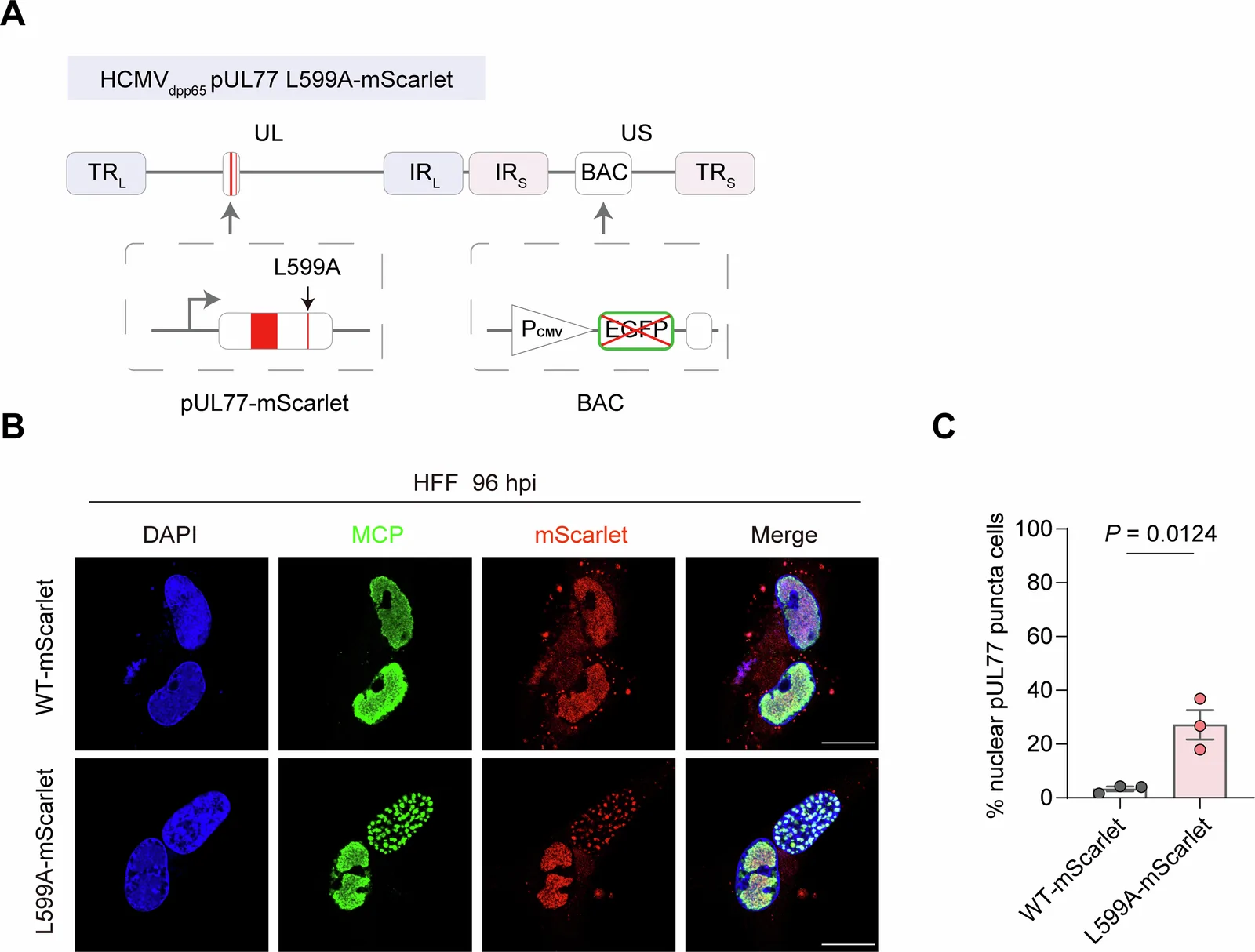
**The mutation L599A disrupts pUL77 nuclear export.** **(A)** Schematic genome diagram of HCMV_dpp65_ pUL77 L599A-mScarlet. **(B)** Subcellular distribution of pUL77 L599A-mScarlet. HFFs were infected with HCMV_dpp65_ pUL77-mScarlet (WT-mScarlet) or the mutant (L599A-mScarlet). At 96 hpi, the localization of pUL77-mScarlet (red) and MCP (green) was detected by confocal microscopy. Nuclei were counterstained with DAPI (blue). Scale bars, 20 μm. **(C)** Quantitative analysis of nuclear pUL77 puncta. The percentage of cells exhibiting nuclear pUL77 punctate was quantified (>100 cells per group). Data were collected from three independent experiments, and results are presented as mean ± SEM. *P* value was calculated using a two-tailed unpaired *t*-test.

**Fig. S6:**
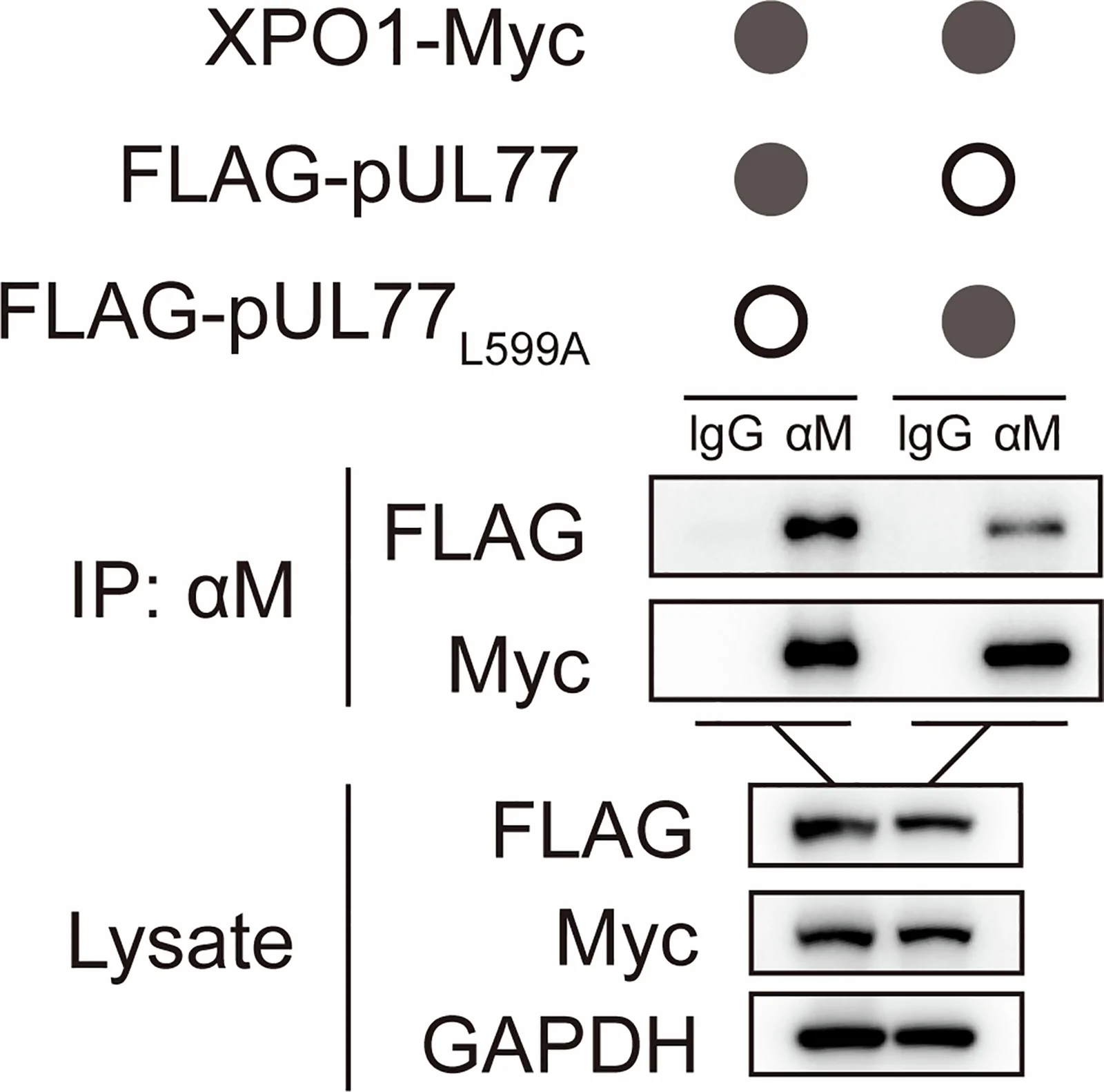
**The pUL77_L599A_ point mutation attenuates the interaction between pUL77 and XPO1.** 293T cells were co-transfected with constructs expressing Myc-XPO1 and FLAG-tagged pUL77 or pUL77_L599A_. At 48 h post-transfection, cells were harvested and cell lysates were subjected to immunoprecipitation with an anti-Myc monoclonal antibody (αM) or control IgG, followed by immunoblotting with the indicated antibodies.

**Fig. S7:**
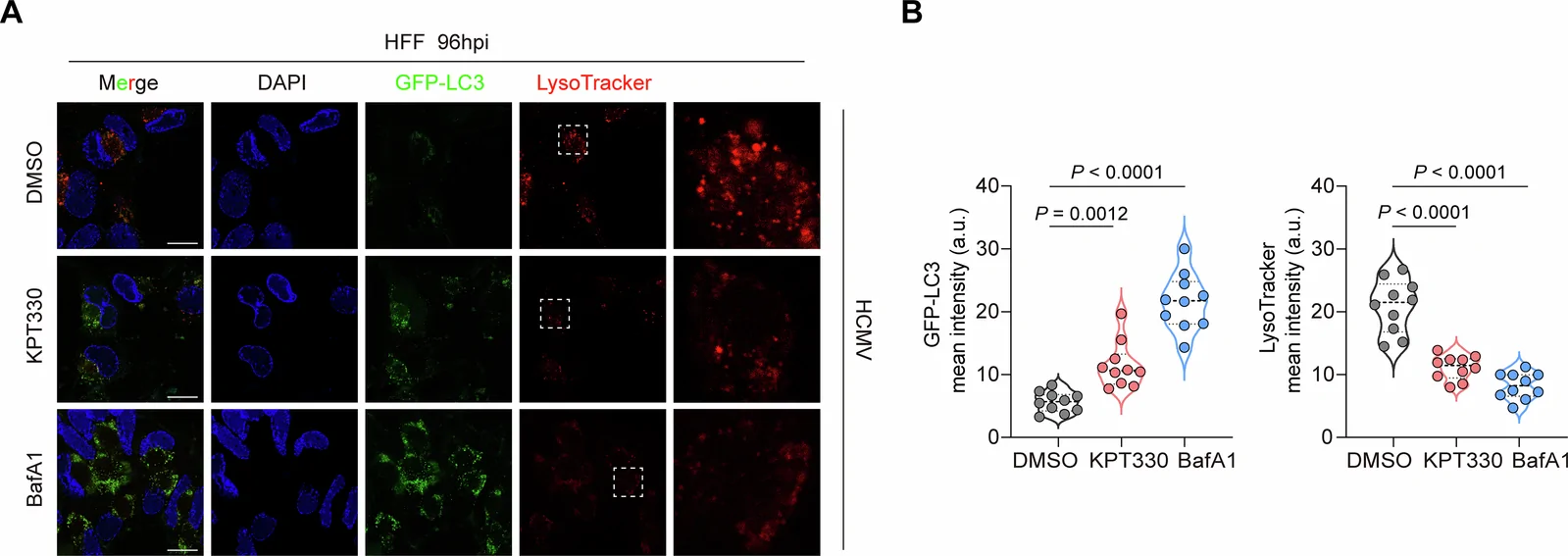
**KPT330 inhibits lysosomal acidification.** **(A)** Stable GFP-LC3 HFF cell lines were infected with HCMV and treated with DMSO or KPT330 (20 nM). BafA1 (20 nM) was added at 48 hpi and maintained for 48 h. At 96 hpi, cells were co-stained with LysoTracker Red DND-99 (50 nM) to label lysosomes and Hoechst 33342 to label nuclei for 1 h, followed by live-cell imaging on a Leica confocal microscope. The white dashed box is enlarged in the right panel. Scale bars, 20 μm. **(B)** Mean fluorescence intensities of GFP-LC3 and LysoTracker in ten random fields. Results are presented as mean ± SD, and *P* values were calculated by one-way ANOVA with Dunnett's T3 multiple-comparisons test.

## Appendix A Supplementary data

Table S1 Predicted candidate NES motifs, Related to Fig. 4.

Table S2 Primers for viruses and plasmids, and shRNA sequences.

Table S3 Antibody information.

Table S4 Summary of confidence metrics for structural predictions.
